# Identification of key genes related to arginine metabolism in immunoglobulin A nephropathy through the combination of scRNA-seq and bulk RNA-seq data

**DOI:** 10.3389/fimmu.2026.1888069

**Published:** 2026-08-07

**Authors:** Hong Xia, Wenze Jiang, Mengting Zhu, Yubing Li, Shengcheng Tai, Dandan Qiu, Zhenliang Fan, Zhejun Chen, Yan Liu, Peipei Zhang, Keda Lu

**Affiliations:** 1 Zhejiang Chinese Medical University, Hangzhou, China; 2 Nephrology Department, The First Affiliated Hospital of Zhejiang Chinese Medical University, Hangzhou, China; 3 Nephrology and Rheumatology Department, The Third Affiliated Hospital of Zhejiang Chinese Medical University, Hangzhou, China

**Keywords:** immunoglobulin A nephropathy, arginine metabolism, biomarker, single-cell analysis, RNA‑seq

## Abstract

**Background:**

Immunoglobulin A nephropathy (IgAN) is a major cause of chronic kidney disease and kidney failure. Currently, arginine metabolism (AM)-related genes (AMRGs) have been reported to play direct or indirect roles in various kidney-related diseases. This study aimed to identify AM-associated key genes in IgAN, potentially paving the way for targeted therapies.

**Methods:**

The data pertaining to IgAN and AMRGs were procured from public databases and literature, respectively. Candidate genes were obtained by integrating differentially expressed genes (DEGs) with AMRGs, and the genes were experimentally verified using qPCR. The identification of key genes was facilitated by machine learning algorithms and gene expression analyses. Of particular significance was the use of the nomogram to evaluate the diagnostic efficacy of these key genes. Functional enrichment, immune infiltration, drug prediction, and molecular docking analyses were performed. Single-cell analysis was employed to ascertain cell types, with the identification of key cells facilitated by key genes.

**Results:**

ARG1 and ARG2 were identified as key genes, and the expression of these 2 genes was found to be downregulated in IgAN samples. The nomogram developed utilizing these key genes demonstrated a satisfactory capacity for differentiating among various sample types. The key genes were found to be enriched in several signaling pathways, including PIP3 signaling in B lymphocytes and BCR signaling pathway. Furthermore, most of the differential immune cells showed significant negative correlations with key genes. Effector memory CD8+ T cells exhibited extremely significant negative correlations with both ARG1 and ARG2 (correlation coefficient (r) < -0.70, P < 0.001). Riluzole exhibited strong binding affinity for key genes and formed stable complexes. Finally, monocytes were considered key cells and played a critical role in IgAN.

**Conclusion:**

This study suggests that ARG1 and ARG2 may be key genes and potential mechanistic indicators of IgAN associated with AM, but it needs to be further verified in non-invasive samples and larger independent cohorts. Additionally, monocytes were recognized as key cells in the progression of IgAN, providing valuable insights to support the development of targeted therapies.

## Graphical Abstract

**Figure:**
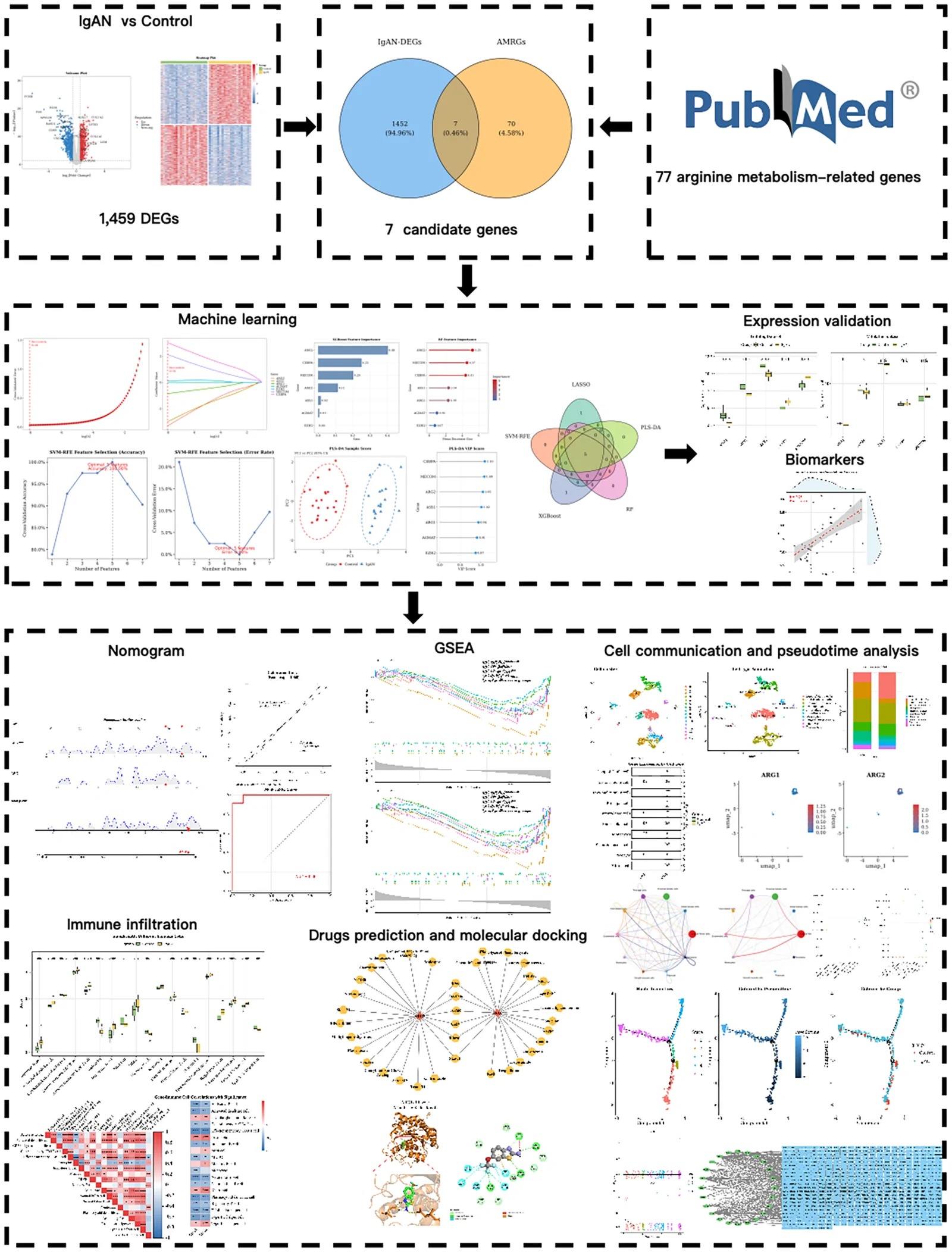

## 1 Introduction

Immunoglobulin A nephropathy (IgAN) is the most common form of primary glomerulonephritis worldwide. Patients have a higher lifetime risk of kidney failure, with approximately 40% progressing to end-stage renal disease (ESRD) within 20 years (1). The clinical presentation of IgAN ranges from asymptomatic microscopic or intermittent gross hematuria with stable renal function to rapidly progressive glomerulonephritis (2). The pathogenesis of IgAN is complex and has not yet been fully elucidated. The currently prevailing hypothesis suggests that the development of IgAN involves a “four-hit process,” the core of which lies in galactose-deficient IgA1 (Gd-IgA1) resulting from genetic susceptibility and the ensuing cascade of immune inflammatory responses (3). The early symptoms of IgAN are insidious, and definitive diagnosis relies on renal biopsy; however, renal biopsy is an invasive procedure carrying the risk of infection. Therefore, the development of early diagnostic key genes is of great significance for exploring molecular mechanisms, developing accurate diagnostic models, and promoting early detection and treatment (4).

In recent years, some components involved in the pathogenesis of IgAN, such as autoantibodies against Gd-IgA1 and microRNAs, have been developed as key genes for the diagnosis of the disease (5, 6). Arginine metabolism (AM) is closely related to the pathogenesis of IgAN through multiple interrelated mechanisms. L-arginine is mainly metabolized through two competing pathways: the inducible nitric oxide synthase (iNOS) pathway and the arginase pathway (7). These pathways regulate the bioavailability of nitric oxide, endothelial function, oxidative stress, immune cell activation, and extracellular matrix deposition (8). Therefore, the imbalance of AM may participate in the progression of IgAN by affecting the immune response related to B cells, inflammatory injury, and renal fibrotic remodeling (9). ARG1 and ARG2 are key enzymes in the arginase pathway and have been considered to be involved in immune regulation, kidney injury, oxidative stress, and fibrosis. However, their expression patterns and potential value as biomarkers in IgAN remain unclear. Importantly, genetic evidence further supports the role of AM in IgAN (10, 11). A large-scale meta-analysis of genome-wide association studies (GWAS) targeting genetic susceptibility loci of IgAN identified peptidyl arginine deiminase 4 (PADI4) - an enzyme that converts arginine residues to citrulline residues - as a strong candidate gene for IgAN. PADI4 may be involved in the generation of granulocytes and macrophages, thereby triggering the inflammation and immune response associated with IgAN. In summary, these evidence indicates that AM is at the intersection of the three key pillars of the IgAN pathogenesis - B cell activation, immune regulation, and renal inflammation (12). Therefore, studying the role of AMRGs in IgAN is helpful in elucidating the molecular mechanism of IgAN and promoting the development of key genes with diagnostic significance.

Single-cell RNA sequencing (scRNA-seq) is an emerging high-throughput sequencing technology that enables the detection of cellular heterogeneity within tissues at single-cell resolution (13), Compared to traditional high-throughput RNA sequencing, this method offers significant advantages in studying cellular diversity and the transcriptome within complex tissues. Currently, when combined with bioinformatics methods, scRNA-seq is widely used for disease diagnosis and research into the role of metabolites in various diseases (14–16). However, data integrating single-cell and RNA sequencing to explore the pathology of IgAN remains limited.

This study utilized IgAN bulk RNA-seq and scRNA-seq data from public databases to identify key genes associated with AM in IgAN and employed a series of bioinformatics analysis methods to investigate the mechanisms underlying these key genes in IgAN. Cell types were annotated via single-cell analysis to clarify the expression distribution of key genes across different cell populations, providing novel insights into the arginine metabolism mechanism in IgAN and targeted therapeutic strategies.

## 2 Materials and methods

### 2.1 Data source

The data of immunoglobulin A nephropathy (IgAN) were obtained from the Gene Expression Omnibus (GEO) database (https://www.ncbi.nlm.nih.gov/geo/). The GSE93798 dataset (platform: GPL22945) was employed as the training set, which included 20 IgAN glomerular biopsy tissue samples and 22 control glomerular biopsy tissue samples obtained from healthy donors (non-cancerous kidney tissues from living donors or transplant controls). The GSE35487 dataset (platform: GPL96) served as the validation set, comprising 25 IgAN tubulointerstitial biopsy tissue samples and 6 control tubulointerstitial biopsy tissue samples from healthy donors. The single-cell RNA sequencing (scRNA-seq) dataset GSE171314 (platform: GPL20301) contained 4 IgAN renal biopsy tissue samples and 1 control renal biopsy tissue sample from a healthy donor. In addition, a total of 77 arginine metabolism-related genes (AMRGs) were retrieved from the literature (9). (**Supplementary Table 1**). This gene set encompasses key enzymes and transporters involved in arginine biosynthesis, catabolism, and the urea cycle. We adopted this published set directly to ensure reproducibility and avoid selection bias.

### 2.2 Differential expression analysis

To identify differentially expressed genes (DEGs) between IgAN and control groups, differential expression analysis was performed using the limma package (v 3.56.2) (17) on the training set (|log_2_ fold change (FC)| > 0.5, P < 0.05). Moreover, the ggplot2 package (v 3.5.1) (18) was used to generate a volcano plot of DEGs, with the top 10 significantly upregulated and downregulated genes (sorted by |log_2_ FC| in descending order) labeled. A heatmap of these genes was then generated using the pheatmap package (v 1.0.12) (19).

### 2.3 Identification, functional enrichment, and protein-protein interaction network of candidate genes

To identify the genes associated with AM in IgAN, the ggvenn package (v 0.1.10) (20) was employed to determine the intersection of DEGs and AMRGs. These genes were subsequently documented as candidate genes for further analysis. Based on the candidate genes, Gene Ontology (GO) and Kyoto Encyclopedia of Genes and Genomes (KEGG) analyses were conducted to explore the biological functions and pathways associated with these genes, utilizing the clusterProfiler package (v 4.15.0.003) (21). A significance threshold of adjusted P < 0.05 was applied. Notably, the analyzed GO terms included biological process (BP), cellular component (CC), and molecular function (MF). The candidate genes were submitted to the Search Tool for the Retrieval of Interacting Genes/Proteins (STRING) database (https://string-db.org/) with an interaction score threshold set above 0.15 to explore their protein-level interactions. These interactions were then visualized using Cytoscape software (v 3.10.3) (22).

### 2.4 Machine learning and expression validation

For identifying feature genes, candidate genes were subjected to machine learning and expression validation analyses in the training set. Specifically, glmnet package (v 4.1-8) (23)was employed to perform Least Absolute Shrinkage and Selection Operator (LASSO) regression of these genes, and the best lambda value that minimized the error was determined via 5-fold cross-validation to screen for LASSO genes. Support Vector Machine-Recursive Feature Elimination (SVM-RFE) algorithm was applied to these genes using the caret package (v 6.0-94) (24), which was employed to screen for the most optimal SVM-RFE genes based on 5-fold cross-validation (with a radial basis function kernel (sigma = 0.8, C = 0.6), selecting the top 5 features with the highest cross-validated accuracy). The XGBoost package (v 2.1.1.1) (25) was used to analyze candidate genes. The core parameter settings included a binary classification logistic regression objective function (objective = “binary: logistic”), a log loss evaluation metric (eval_metric = “logloss”), a maximum tree depth of 3, a learning rate of 0.1, a leaf node splitting threshold of 0.1, sample and feature sampling rates both of 0.8, and 50 iterations. Genes with non-zero feature importance (Gain) were extracted as the screening threshold to obtain eXtreme Gradient Boosting (XGBoost) genes. When analyzing candidate genes using the randomForest package (v4.7-1.2) (26), the core parameter was set to 1000 trees. The Top 5 genes with high importance were extracted as the screening threshold by sorting according to the Mean Decrease Gini value, and finally the Random Forest (RF) genes were obtained. When performing Partial Least Squares Discriminant Analysis (PLS-DA) using the mixOmics package (v 6.24.0) (27), the core parameter settings were as follows: the response variable was a binary variable (where “1” represented the disease group and “0” represented the control group); 5-fold cross-validation was adopted (the “validation” parameter was set to “Mfold” and the “folds” parameter was set to 5), with 5 independent repetitions (the “nrepeat” parameter was set to 5); and the Balanced Error Rate (BER) was used as the evaluation metric. The model determined the optimal number of principal components through cross-validation, and then screened out the top 5 genes with the highest scores based on Variable Importance in Projection (VIP) scores, which were defined as PLS-DA genes. To ensure the reproducibility of all stochastic processes, a fixed random seed (set.seed(123)) was applied across all algorithms. Importantly, all feature selection and model training were performed exclusively on the training set (GSE93798). Cross-validation performance evaluation was also conducted within the training set, while the independent validation set (GSE35487) was reserved solely for validating the expression differences and diagnostic generalizability of the identified biomarkers and was not involved in any stage of model training or feature selection. Furthermore, the VennDiagram package (v 1.7.3) (20) was applied to obtain the intersection of genes identified by the 5 algorithms, which were defined as feature genes. An investigation was conducted to compare the expression of feature genes in the IgAN and control groups, using the GSE93798 and GSE35487 datasets. The Wilcoxon test was employed to analyse the datasets (P < 0.05). Genes exhibiting consistent expression trends and significant inter-group differences in both datasets were identified as key genes. To analyze the correlations among key genes, Spearman’s analysis from the psych package (v 2.4.6.26) (28) was used to determine the correlations between key genes in all samples of the training set (|correlation coefficient (r)| > 0.30, P < 0.05).

### 2.5 Developing and assessing a nomogram

Within the confines of the training set, a nomogram model was formulated, underpinned by key genes, utilising the rms package (v 6.8-1) (29) in order to determine the likelihood of the occurrence of IgAN. To assess the predictive accuracy of the nomogram, a calibration curve was generated by regplot package (v 1.1) (30), and the Hosmer-Lemeshow (HL) test was conducted to assess the fit between predicted values and actual outcomes (P > 0.05). The ROC curve was deployed to ascertain the efficacy of the nomogram in a clinical setting via pROC package (v 1.18.5) (31), with a diagnostic effect of an area under the curve (AUC) exceeding 0.7 serving as a valuable reference point. Additionally, the same nomogram model was constructed and evaluated in the validation cohort to further assess its generalizability.

### 2.6 GeneMANIA network, gene set enrichment analysis, and gene set variation analysis

To gain deeper insights into the interactions between key genes and their associated biological functions, a gene co-expression network was constructed using the GeneMANIA website (http://www.genemania.org/). The GSEA was performed to elucidate the biological functions of key genes during IgAN progression. The reference gene set (c2.cp.all.v2022.1.Hs.symbols.gmt) was retrieved from the Molecular Signatures Database (MSigDB, https://www.gsea-msigdb.org/gsea/msigdb). The initial step was to calculate the Spearman correlation coefficients between each biomarker and each gene across all samples from the training set, using the stats package (v 4.3.3) (32). Subsequently, genes were ranked in descending order based on their Spearman correlation coefficients with each biomarker, and this ranking was used as the test gene set for subsequent GSEA. Subsequently, GSEA was performed using the clusterProfiler package (v 4.15.0.003) with the following thresholds: |normalized enrichment score (NES)| > 1, P < 0.05, and false discovery rate (FDR) < 0.25.

To further investigate the biological functional differences between IgAN and control groups, GSVA was performed. Specifically, the reference gene set (h.all.v7.4.symbols.gmt) was downloaded from MSigDB database. Of the training set, GSVA score for each sample was calculated using the single-sample gene set enrichment analysis (ssGSEA) algorithm from the GSVA package (v 1.53.28) (33). The limma package (v 3.56.2) was then applied to compare the differences in GSVA scores between IgAN and control groups, with the thresholds established at |t| > 2 and P < 0.05.

### 2.7 Immune infiltration analysis

The single-sample gene set enrichment analysis (ssGSEA) algorithm, as implemented within the GSVA package (v 1.53.28), was employed to calculate the infiltration scores of 28 immune cell types (34) for each sample in the training set. Moreover, the Wilcoxon test was conducted to identify immune cells exhibiting notable disparities between IgAN and control groups (P < 0.05). Subsequently, the psych package (v 2.4.6.26) was used to analyze the correlations among the significantly different immune cells and between the key genes and the significantly different immune cells (|r| > 0.30, P < 0.05).

### 2.8 Regulatory network construction, drug prediction and molecular docking

To further explore the upstream regulatory mechanism of key genes, a TF-mRNA regulatory network was constructed. The TFs that regulated the key genes were predicted using the JASPAR database (https://www.networkanalyst.ca/NetworkAnalyst/Secure/AnalysisOverview.xhtml) in the NetworkAnalyst database (https://www.networkanalyst.ca/). The resulting TF-mRNA interaction network was visualized using Cytoscape software (v 3.10.3).

To identify drugs that interacted with the key genes, potential drugs targeting these genes were screened using the drug-gene interaction database (DGIdb, http://dgidb.genome.wustl.edu/). The resulting biomarker-drug interaction network was visualized using Cytoscape software (v 3.10.3). In this study, drugs that interacted simultaneously with key genes and were approved for marketing by the FDA were selected as the key drugs for molecular docking. The molecular structure of the drug was downloaded from PubChem (https://pubchem.ncbi.nlm.nih.gov/), and the three-dimensional structures of the biomarker proteins were retrieved from the PDB (https://www.rcsb.org/structure/) database. The PyMOL software (v 3.1.4) (35) was used to remove water molecules and original ligands, and convert the SDF file of the drug into a PDB file. Subsequently, the AutoDockTools software (v 1.5.7) (36) was employed to convert the PDB files of proteins and small molecules into the pdbqt format. AutoDock Vina software (v 1.2.3) (37) was utilized for molecular docking to calculate and output the binding energy; a binding energy lower than -5 kcal/mol usually indicates strong intermolecular or intramolecular interactions, which have significant biological significance.

### 2.9 Treatment of scRNA-seq data and selection of key cells

In the GSE171314 dataset, the Seurat package (v 5.1.0) (38) was utilized for quality control (quality control criteria: 200 < nFeature_RNA < 4,000, 500 < nCount_RNA < 15,000). For the filtered data, the NormalizeData function was used to standardize it and obtain the normalized data. Then the FindVariableFeatures function was used to extract the top 2,000 highly variable genes (HVGs) for subsequent analyses (the 10 HVGs with the largest variation were labeled). Principal component analysis (PCA) was conducted on the top 2,000 HVGs via runPCA function. Subsequently, from the results obtained via the ElbowPlot function, the top 30 principal components (PCs) were chosen (P < 0.05). Subsequently, the resolution parameter was configured to 0.5, and an unsupervised clustering analysis was carried out on every cell within the dataset. Then, the outcomes of the clustering were visualized through the application of the uniform manifold approximation and projection (UMAP) algorithm. Cell types of the different clusters were identified after dimensionality reduction and clustering, employing the reference (39) and CellMarker database (http://www.bio-bigdata.center/CellMarkerSearch.jsp). The annotation results were visualized on a UMAP plot, and the expression of marker genes in different cell types was depicted. Histograms were utilized to illustrate the proportions of various cell types in the all samples.

The Wilcoxon test was applied to compare the expression differences of key genes between IgAN samples and control samples across differential cells (P < 0.05). Subsequently, the distribution of key genes in annotated cells were observed in the GSE171314 dataset. Cells with differential expression of key genes and high expression distribution were defined as key cells.

### 2.10 Cell communication, pseudotime, and transcriptional regulation factors analyses

To investigate the communication network among annotated cells, the CellChat package (v 1.6.1) (40) was used to analyze the communication relationships in the GSE171314 dataset and to establish a communication network. Subsequently, the CellChat package (v 1.6.1) was utilized to identify ligand-receptor (L-R) interactions between key cells and other cells. Meanwhile, in order to identify the differentiation process of key cells and the expression of key genes at different differentiation stages, the UMAP clustering method was used to perform dimensionality reduction and clustering on the key cells (resolution = 0.2). Then, the monocle package (v 2.28.0) (41) was used for pseudotime analysis. The expression changes of key genes throughout the pseudotime progression were also analyzed.

To dissect the core transcriptional regulatory mechanisms of key cells, further identify their upstream regulatory factors, and unravel the gene regulatory pathways, this study constructed a TF-target gene regulatory network based on the SCENIC software (v 1.3.1) (42). Gene filtering was performed on the expression matrix of key cell subsets, retaining genes expressed in at least 3% of cells with counts greater than 1 for subsequent network construction analysis. The GEne Network Inference with Ensemble of Trees (GENIE3) algorithm was used to construct a co-expression matrix, and high-confidence co-expressed TF-target relationships were screened. The RcisTarget module was applied, combined with the hg19 species database (https://resources.aertslab.org/), to conduct motif enrichment analysis. The AUCell package (v 0.3.1) (43) was used to calculate the transcriptional factor activity scores of key cell subsets (AUC threshold: Top 5% genes). After obtaining high-confidence regulons, the core axis of transcriptional regulation was identified, and the relevant data were imported into Cytoscape software (v 3.10.3) to draw the regulatory network diagram.

### 2.11 Patient enrollment and clinical samples

Renal tissue samples were collected from patients treated at The First Affiliated Hospital of Zhejiang Chinese Medical University from October 2025 to October 2026. This study was approved by the Ethics Committee of The First Affiliated Hospital of Zhejiang Chinese Medical University(Approval No. 025-KLS-561-03), and written informed consent was obtained from all participants or their legal representatives before sample collection.

The IgAN group included patients who were diagnosed with primary IgA nephropathy by renal biopsy. The diagnosis was established based on clinical manifestations, light microscopy, immunofluorescence, and/or electron microscopy findings, with dominant or co-dominant IgA deposition in the glomerular mesangial area. Patients with secondary IgAN, including IgA vasculitis, systemic lupus erythematosus, chronic liver disease, diabetic nephropathy, malignancy-associated nephropathy, or other secondary glomerular diseases, were excluded. Patients who had received corticosteroids or immunosuppressive therapy before renal biopsy were also excluded when applicable.

The control group consisted of adjacent non-tumorous renal tissues obtained from patients who underwent nephrectomy for renal carcinoma. Control renal tissues were collected from areas macroscopically distant from the tumor margin and were confirmed by pathological examination to be free of tumor infiltration. Patients with a history of chronic kidney disease, autoimmune disease, primary glomerular disease, or systemic inflammatory disease were excluded from the control group.

Finally, five renal tissue samples from patients with biopsy-proven IgAN and five adjacent non-tumorous renal tissue samples from renal carcinoma patients were included for RT-qPCR validation. Basic clinical information, including age, sex, serum creatinine, estimated glomerular filtration rate, urinary protein excretion, and pathological findings, was collected from medical records when available.

### 2.12 Sample collection and preservation

For IgAN patients, renal biopsy tissue was obtained under ultrasound guidance according to standard clinical procedures. A small portion of renal tissue available for molecular analysis was immediately rinsed with pre-cooled phosphate-buffered saline to remove residual blood. For control subjects, adjacent non-tumorous renal tissue was collected immediately after surgical resection. All tissue samples were rapidly snap-frozen in liquid nitrogen and stored at −80 °C until RNA extraction. Repeated freeze–thaw cycles were avoided.

Total RNA was extracted from renal tissue samples using the SteadyPure RNA Extraction Kit (Accurate Biology, Cat. No. AG21024) in accordance with the manufacturer’s protocol. Briefly, approximately 20–30 mg of frozen renal tissue was homogenized in the lysis buffer supplied with the kit under RNase-free conditions. The lysate was then processed using the spin-column purification system provided in the kit. After RNA binding to the column membrane, the column was washed sequentially with the recommended washing buffers to remove proteins, genomic DNA, salts, and other contaminants. Finally, total RNA was eluted with RNase-free water.

The concentration and purity of total RNA were measured using a spectrophotometer. RNA samples with an A260/A280 ratio of approximately 1.8–2.0 were used for reverse transcription. All RNA samples were stored at −80 °C until further RT-qPCR analysis.

### 2.13 Reverse transcription and quantitative real-time PCR

Complementary DNA was synthesized from total RNA using a reverse transcription kit according to the manufacturer’s protocol. Briefly, 1 μg of total RNA from each sample was reverse-transcribed into cDNA in a final reaction volume of 10 μL. The synthesized cDNA was stored at −20 °C until quantitative real-time PCR analysis.

Quantitative real-time PCR was performed using SYBR Green PCR Master Mix on a real-time PCR detection system. Each reaction was performed in a final volume of 20 μL containing SYBR Green Master Mix, forward and reverse primers, diluted cDNA template, and nuclease-free water. The amplification conditions were as follows: initial denaturation at 95 °C for 30 s, followed by 40 cycles of denaturation at 95 °C for 5 s and annealing/extension at 60 °C for 30 s. A melting curve analysis was performed to confirm the specificity of amplification. Each sample was tested in triplicate.

The mRNA expression levels of ARG1 and ARG2 were normalized to the internal reference gene GAPDH. The primer sequences used in this study are listed in **Supplementary Table 7**.

### 2.14 Data analysis

The cycle threshold values were obtained from the real-time PCR system. The relative expression levels of ARG1 and ARG2 were calculated using the 2−ΔΔCt method. Briefly, ΔCt was calculated as the Ct value of the target gene minus the Ct value of the internal reference gene. ΔΔCt was calculated by comparing the ΔCt value of each IgAN sample with the mean ΔCt value of the control group. Relative mRNA expression was then expressed as 2−ΔΔCt.

Statistical analyses were performed using GraphPad Prism. The normality of continuous variables was assessed using the Shapiro–Wilk test. For normally distributed data, comparisons between two groups were performed using an unpaired Student’s t-test. For non-normally distributed data, the Mann–Whitney U test was used. A two-sided P value < 0.05 was considered statistically significant.

## 3 Results

### 3.1 Functional pathways and PPI network of candidate genes

**Figure 1:**
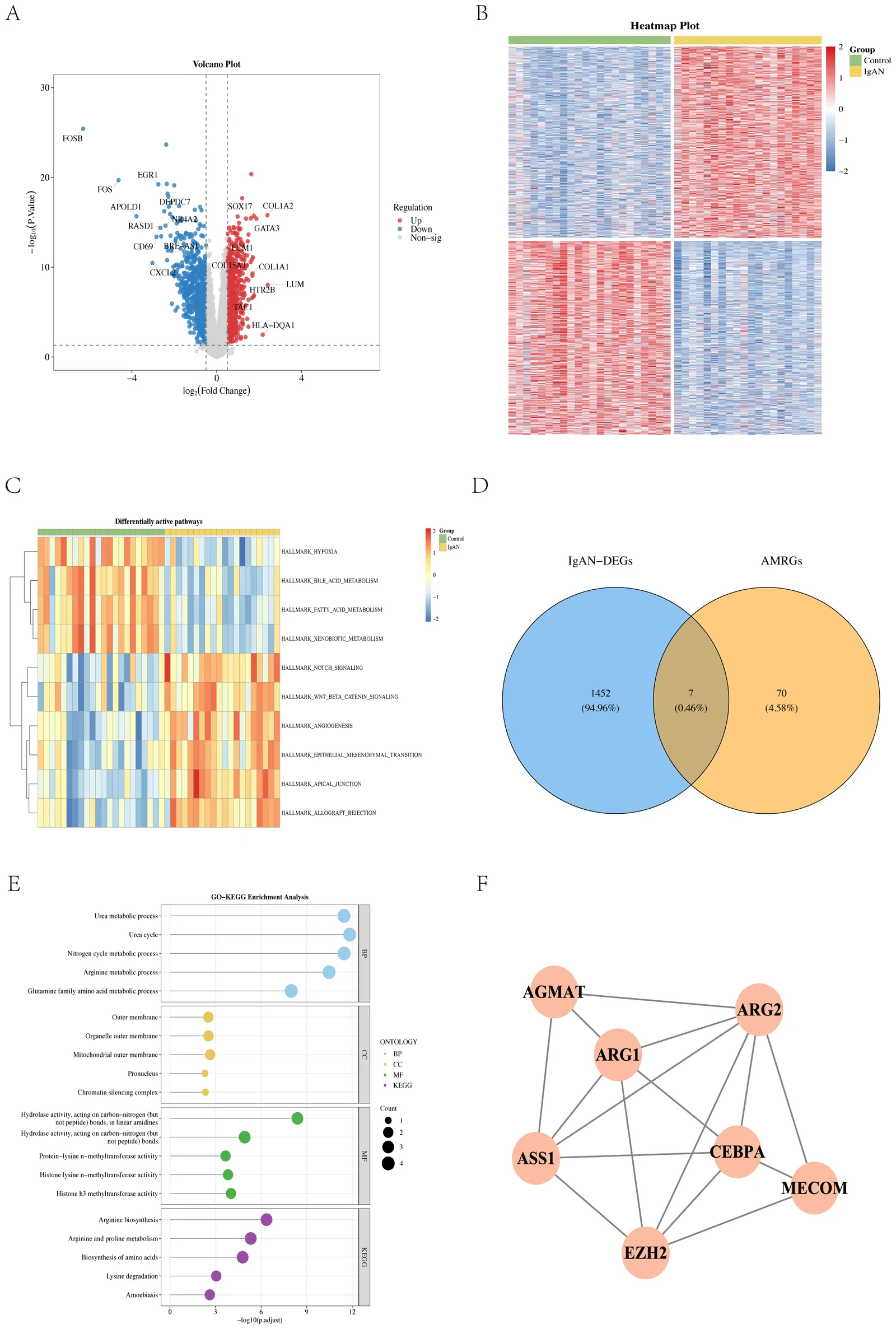
Functional pathways and PPI network of candidate genes. **(A)** Volcano plot showing differentially expressed genes (DEGs) between IgAN and control groups in the GSE93798 training set. Red dots represent upregulated genes, blue dots represent downregulated genes, and gray dots represent genes with no significant differential expression. **(B)** Heatmap displaying the expression patterns of the top 10 significantly upregulated and downregulated genes in IgAN versus control samples. **(C)** GSVA enrichment bar chart showing significantly enriched pathways between IgAN and control groups. The length of each bar represents the enrichment score, with red indicating pathways upregulated in IgAN and blue indicating pathways downregulated in IgAN. **(D)** Venn diagram illustrating the intersection of DEGs and arginine metabolism-related genes (AMRGs), identifying 7 candidate genes for further analysis. **(E)** Bar chart displaying Gene Ontology (GO) functional enrichment (Biological Process, Cellular Component, Molecular Function) and Kyoto Encyclopedia of Genes and Genomes (KEGG) pathway enrichment of the 7 candidate genes. **(F)** Protein-protein interaction (PPI) network of the 7 candidate genes, constructed using the STRING database. Nodes represent proteins, and edges represent known or predicted protein-protein interactions.

Differential expression analysis showed that there were 1,459 DEGs between IgAN and control groups. Among these, 725 genes were upregulated and 734 were downregulated in the IgAN group (**Figures 1A, B**). GSVA analysis identified a total of 33 pathways were identified as significantly enriched (**Figure 1C**; **Supplementary Table 2**). Among these, Notch signaling, Wnt beta catenin signaling, allograft rejection, and epithelial mesenchymal transition were all highly active in IgAN, while bile acid metabolism, fatty acid metabolism, and xenobiotic metabolism exhibited low activity in IgAN. Moreover, a total of 7 shared genes between DEGs and AMRGs were identified and selected as candidate genes (**Figure 1D**). The GO analysis results showed a significant enrichment in the 288 different entries (adjusted P < 0.05) (**Supplementary Table 3**). Among them, 253 BPs like the urea cycle of kinase activity were enriched, 8 CCs like the mitochondrial outer membrane were enriched, and 27 MFs like the histone lysine N-methyltransferase activity were enriched (**Figure 1E**). The KEGG enrichment analysis of the candidate genes revealed that a total of 6 KEGG pathways were enriched, including arginine biosynthesis, arginine and proline metabolism, and biosynthesis of amino acids (adjusted P < 0.05) (**Figure 1E**; **Supplementary Table 4**). Next, a PPI network consisting of 16 interaction relationships corresponding to 7 candidate genes was constructed (**Figure 1F**). In this network, ARG2 exhibited frequent protein-level interactions with other genes.

### 3.2 Identification of ARG1 and ARG2 as key genes

**Figure 2:**
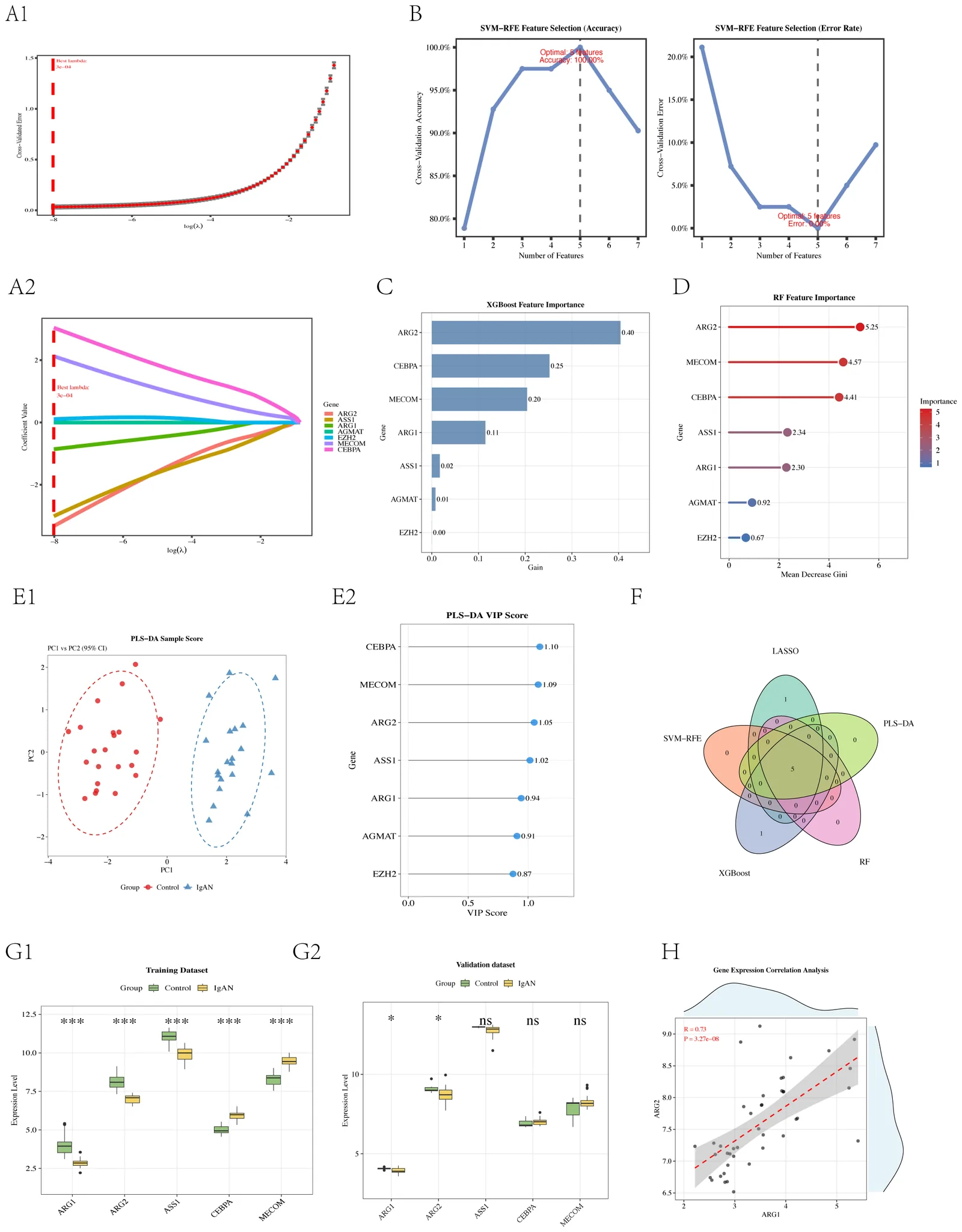
Identification of ARG1 and ARG2 as key genes. LASSO regression analysis of candidate genes in the GSE93798 training set. The optimal lambda value (λ = 0.0003) was determined via 5-fold cross-validation to select LASSO genes. **(B)** Support Vector Machine-Recursive Feature Elimination (SVM-RFE) analysis showing the accuracy and error rates across different numbers of features. The model achieved optimal performance with 5 features. **(C)** eXtreme Gradient Boosting (XGBoost) analysis displaying feature importance scores (Gain) of candidate genes. Genes with non-zero importance were selected as XGBoost genes. **(D)** Random Forest (RF) analysis showing the top 5 genes with the highest Mean Decrease Gini values. **(E)** Partial Least Squares Discriminant Analysis (PLS-DA) showing sample separation between IgAN and control groups. The top 5 genes with the highest Variable Importance in Projection (VIP) scores were selected. **(F)** Venn diagram showing the intersection of genes identified by the five machine learning algorithms (LASSO, SVM-RFE, XGBoost, RF, and PLS-DA), yielding 5 feature genes. **(G)** Expression levels of feature genes (ARG1, ARG2, ASS1, CEBPA, MECOM) in IgAN and control samples from GSE93798 and GSE35487. *P < 0.05, **P < 0.01, ***P < 0.001, ns = not significant. **(H)** Spearman correlation analysis between ARG1 and ARG2 across all samples in the training set (r = 0.73, P < 0.001).

For the LASSO algorithm, when the minimum log lambda value was 0.0003, a total of 6 LASSO genes were screened from the candidate genes (**Figure 2A**). When applying the SVM-RFE algorithm, the model demonstrated the highest prediction accuracy and the lowest error rate with an optimal variable count of 5. This configuration enabled the selection of 5 SVM-RFE genes from the candidate genes: ARG1, ARG2, ASS1, CEBPA, and MECOM (**Figure 2B**). An evaluation of the feature importance assessment results from the XGBoost algorithm was conducted, leading to the selection of 6 XGBoost genes (**Figure 2C**). When the RF model was configured, the resulting model error rate reached its minimum, and the top 5 RF genes were identified based on their importance (**Figure 2D**). PLS-DA score principal component analysis indicated that the 2 groups of samples were clearly separated in the direction of PC1, suggesting that there were significant differences between the control group and the IgAN group in this principal component. In this study, the top 5 genes with VIP scores were extracted and defined as PLS-DA genes (**Figure 2E**). Subsequently, an intersection analysis was performed on the genes identified by the aforementioned machine learning algorithms. This analysis ultimately identified ARG1, ARG2, ASS1, CEBPA, and MECOM as feature genes (**Figure 2F**). Meanwhile, ARG1 and ARG2 were significantly downregulated in the IgAN group (P < 0.05) (**Figure 2G**). Consequently, 2 key genes—ARG1 and ARG2—were selected for subsequent analyses. The investigation revealed a positive correlation between 2 key genes (r = 0.73, P < 0.001) (**Figure 2H**).

### 3.3 Biomarker-based nomogram for IgAN prediction

**Figure 3:**
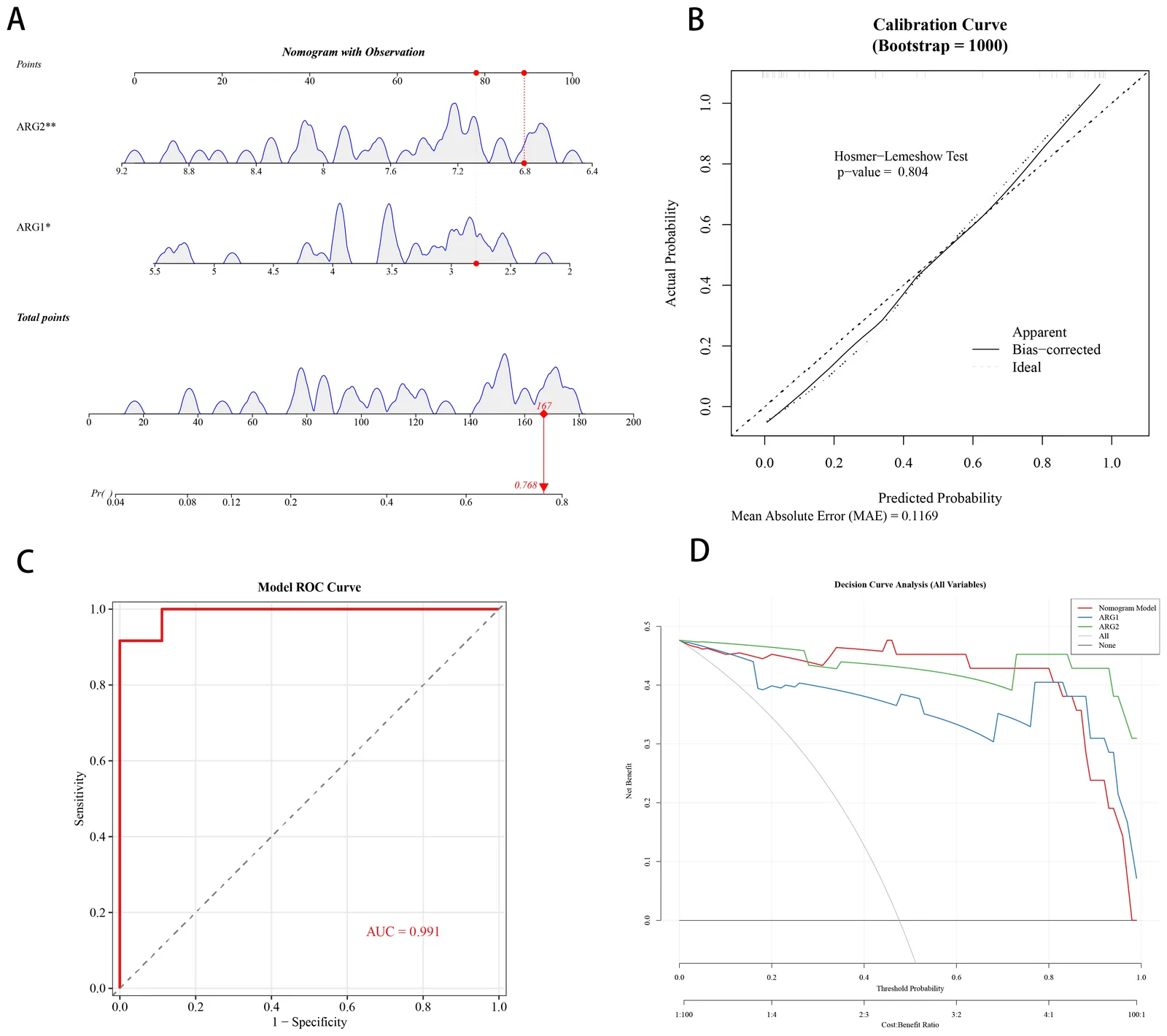
Biomarker-based nomogram for IgAN prediction. **(A)** Nomogram constructed using ARG1 and ARG2 for predicting the risk of IgAN in the GSE93798 training set. Each biomarker is assigned a point value on the top axis; the total points are summed and projected to the bottom axis to estimate the probability of IgAN. **(B)** Calibration curve assessing the predictive accuracy of the nomogram model. The diagonal dashed line represents perfect prediction, while the solid line represents the observed performance of the nomogram. The HL test P-value (P = 0.804) indicates no significant deviation from perfect calibration. **(C)** Receiver operating characteristic (ROC) curve of the nomogram model. The area under the curve (AUC = 0.991) demonstrates excellent discriminatory ability between IgAN and control samples. **(D)** DCA of the nomogram in the training set.The y-axis shows net benefit, and the x-axis shows threshold probability. The red curve represents the nomogram, the gray dashed line represents the “treat-all” strategy, and the black solid line represents the “treat-none” strategy. The nomogram demonstrated superior net clinical benefit across a wide range of threshold probabilities.

A nomogram model was constructed based on the GSE93798 dataset. The model assigned scores corresponding to the expression levels of each biomarker, and the total score was calculated to predict the probability of IgAN occurrence (a total score of 167 corresponded to a disease probability of 0.768) (**Figure 3A**). The calibration curve demonstrated a minimal discrepancy between the actual and predicted risks of IgAN (HL test P = 0.804) (**Figure 3B**). Moreover, the nomogram achieved an AUC of 0.991 (95% CI: 0.973–1.000), indicating high predictive accuracy (**Figure 3C**). DCA revealed that the net benefit of the nomogram was superior to both the “treat-all” and “treat-none” strategies (**Figure 3D**). At the optimal threshold of 0.549 determined by the Youden index, the sensitivity, specificity, and accuracy were 0.917, 1.000, and 0.952, respectively. The positive predictive value (PPV) was 0.571, and the F1 score was 0.704. Calibration was assessed by the Brier score (0.030), indicating good agreement between predicted probabilities and observed outcomes (**Supplementary Table S5**). To further evaluate the generalizability of the model, we applied the same nomogram to the independent validation set GSE35487, which yielded an AUC of 0.862 (95% CI: 0.734–0.990), with a calibration MAE of 0.257 and a HL test P-value of 0.88, indicating satisfactory discrimination and calibration in an external cohort. DCA further confirmed the net clinical benefit of the nomogram in the validation set (**Supplementary Figure 2**). Collectively, these findings confirmed that the nomogram exhibited high predictive accuracy for IgAN risk.

### 3.4 Exploring the biological functions of key genes

**Figure 4:**
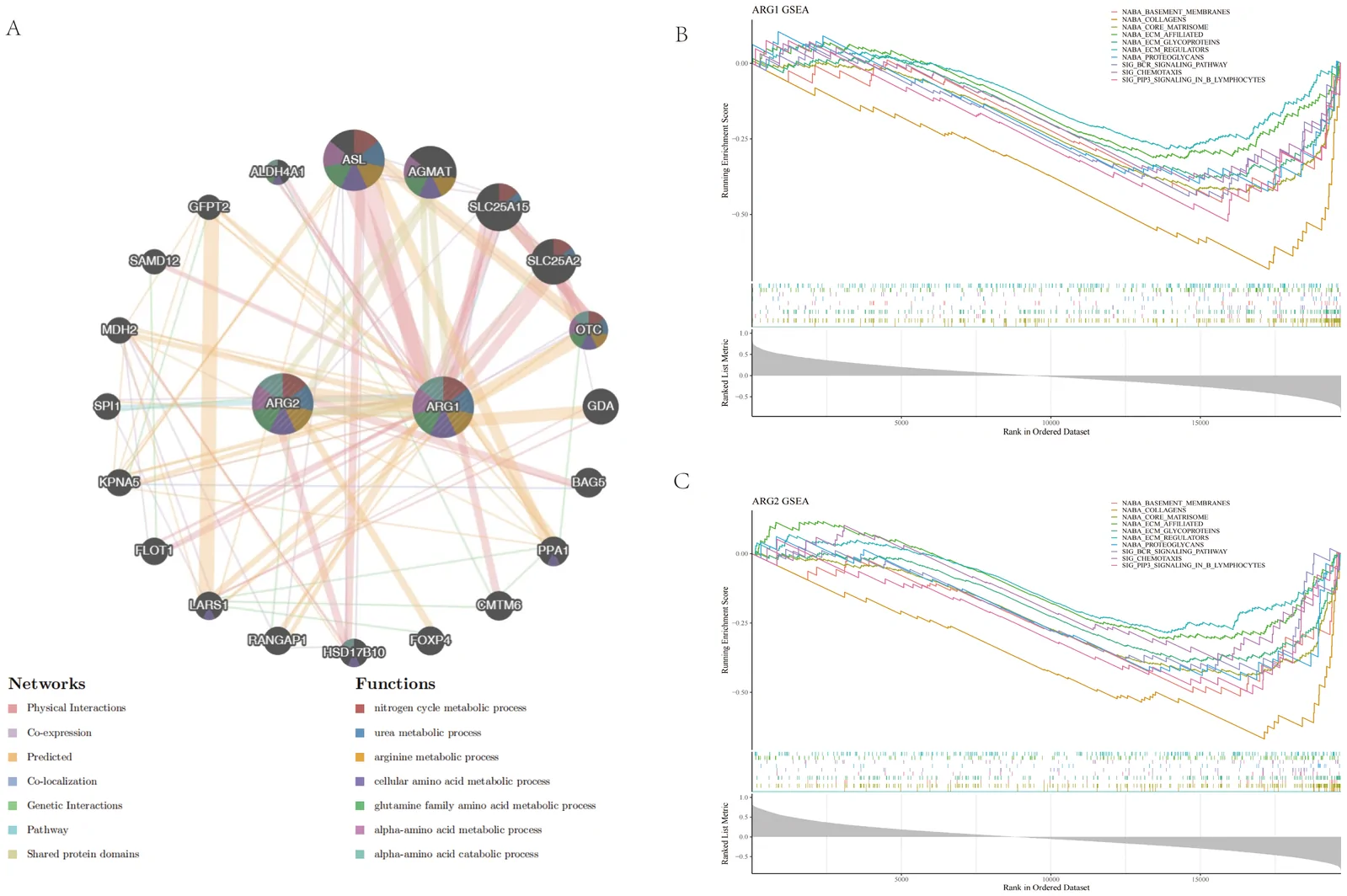
Exploring the biological functions of key genes. **(A)** GeneMANIA network showing the interactions between key genes and their top 20 co-expressed genes. Edge colors represent different interaction types. **(B)** Gene Set Enrichment Analysis (GSEA) for ARG1, showing pathways significantly enriched in correlation with ARG1 expression. The top 8 KEGG pathways are displayed, with the color gradient representing normalized enrichment scores (NES). **(C)** GSEA for ARG2, displaying the top 8 KEGG pathways significantly enriched in correlation with ARG2 expression.

In the GeneMANIA network, key genes interacted with 20 genes and were involved in functions including nitrogen cycle metabolic process, urea metabolic process, and arginine metabolic process (**Figure 4A**). Subsequently, GSEA revealed that 11 and 11 pathways were enriched with respect to the expression of ARG1 and ARG2, respectively (**Supplementary Table 6**). key genes were co-enriched in the PIP3 signaling in B lymphocytes, BCR signaling pathway, ECM affiliated, ECM regulators, and ECM glycoproteins (**Figures 4B, C**). These correlative findings suggest that the biomarkers may be involved in the integration of metabolic homeostasis, immune cell signaling, and extracellular matrix remodeling, offering preliminary clues for understanding their potential biological functions and molecular mechanisms in IgAN. However, further experimental studies are needed to establish the functional significance of these associations.

### 3.5 Strong correlation between key genes and immune cells in IgAN

**Figure 5:**
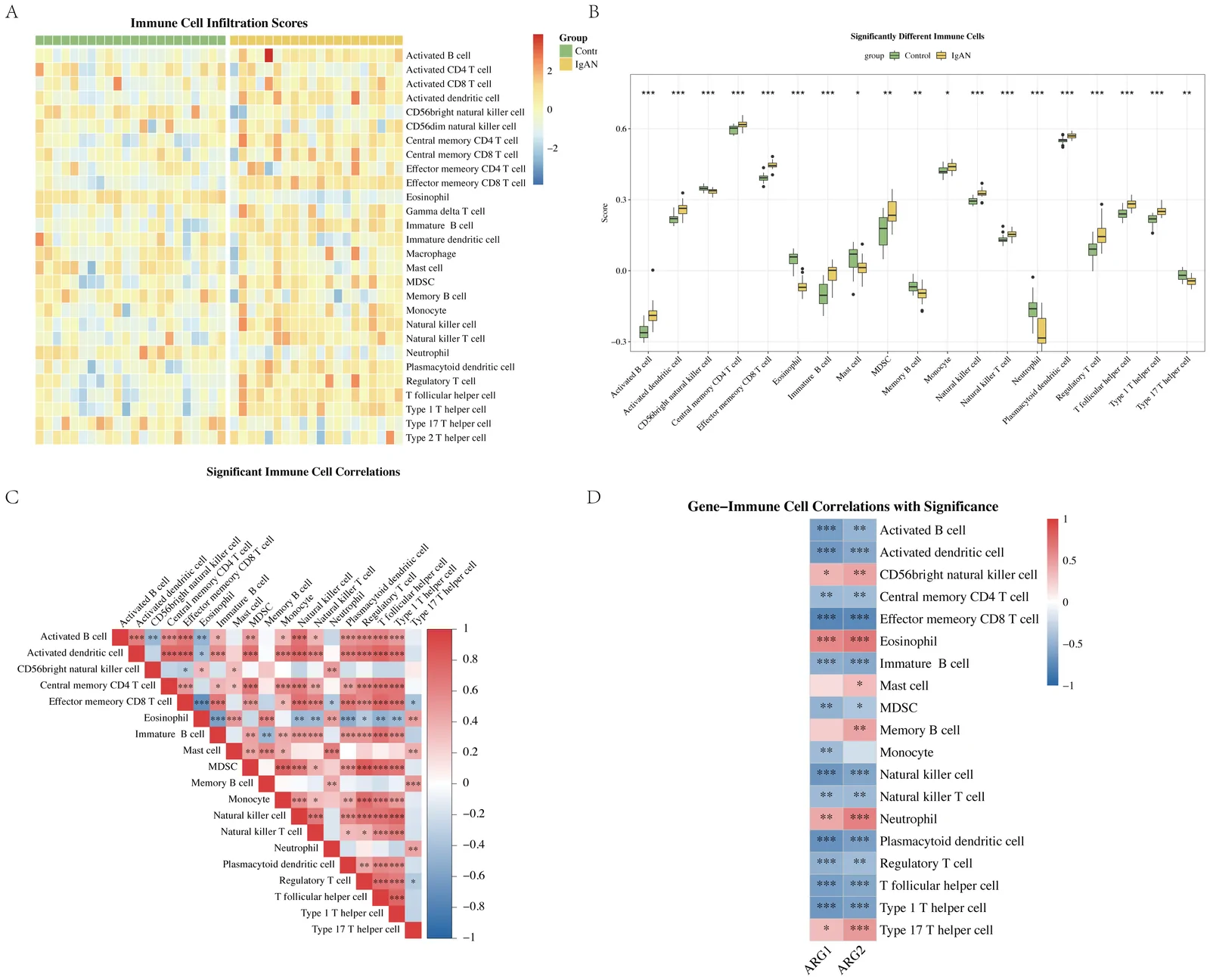
Strong correlation between key genes and immune cells in IgAN. **(A)** Heatmap displaying the infiltration scores of 28 immune cell types in each IgAN and control sample from GSE93798, quantified using the single-sample gene set enrichment analysis (ssGSEA) algorithm.The red indicating high infiltration and blue indicating low infiltration. **(B)** Box plots showing the 28 immune cell types with significantly different infiltration between IgAN and control groups (P < 0.05, Wilcoxon test). **(C)** Correlation heatmap illustrating the pairwise Spearman correlation relationships among the 19 significantly different immune cell types. Red indicates positive correlations, and blue indicates negative correlations. The intensity of the color is proportional to the correlation coefficient. **(D)** Correlation heatmap showing Spearman correlations between key genes (ARG1 and ARG2) and the 19 significantly different immune cells. Red indicates positive correlations, and blue indicates negative correlations. *P < 0.05, **P < 0.01, ***P < 0.001.

The onset of disease is frequently associated with the function of the immune system. Consequently, the examination of immune cell distribution facilitates a more comprehensive investigation of disease pathogenesis. The distribution of immune cell infiltration scores for the 28 immune cell types in the IgAN and control groups was illustrated in the heatmap, which displayed varying degrees of infiltration for each immune cell type (**Figure 5A**). A total of 19 immune cell types exhibited significant differences in immune cell infiltration between the 2 groups (P < 0.05), including activated B cells, monocytes, and neutrophils (**Figure 5B**). Most of these differential immune cells exhibited significant correlations with each other (**Figure 5C**). Among them, the most significant positive correlation was observed between regulatory T cells and myeloid-derived suppressor cells (MDSCs) (r > 0.80, P < 0.001), while the most significant negative correlation was found between effector memory CD8+ T cells and eosinophils (r < -0.60, P < 0.001). Furthermore, most of the differential immune cells showed significant negative correlations with key genes (**Figure 5D**). For example, effector memory CD8+ T cells exhibited extremely significant negative correlations with both ARG1 and ARG2 (r < -0.70, P < 0.001). Additionally, ARG1 and ARG2 also displayed strong positive correlations with eosinophils (r > 0.50, P < 0.001). These findings provided valuable insights into the molecular mechanisms underlying IgAN pathogenesis.

### 3.6 The key genes targeted by TFs and drugs

**Figure 6:**
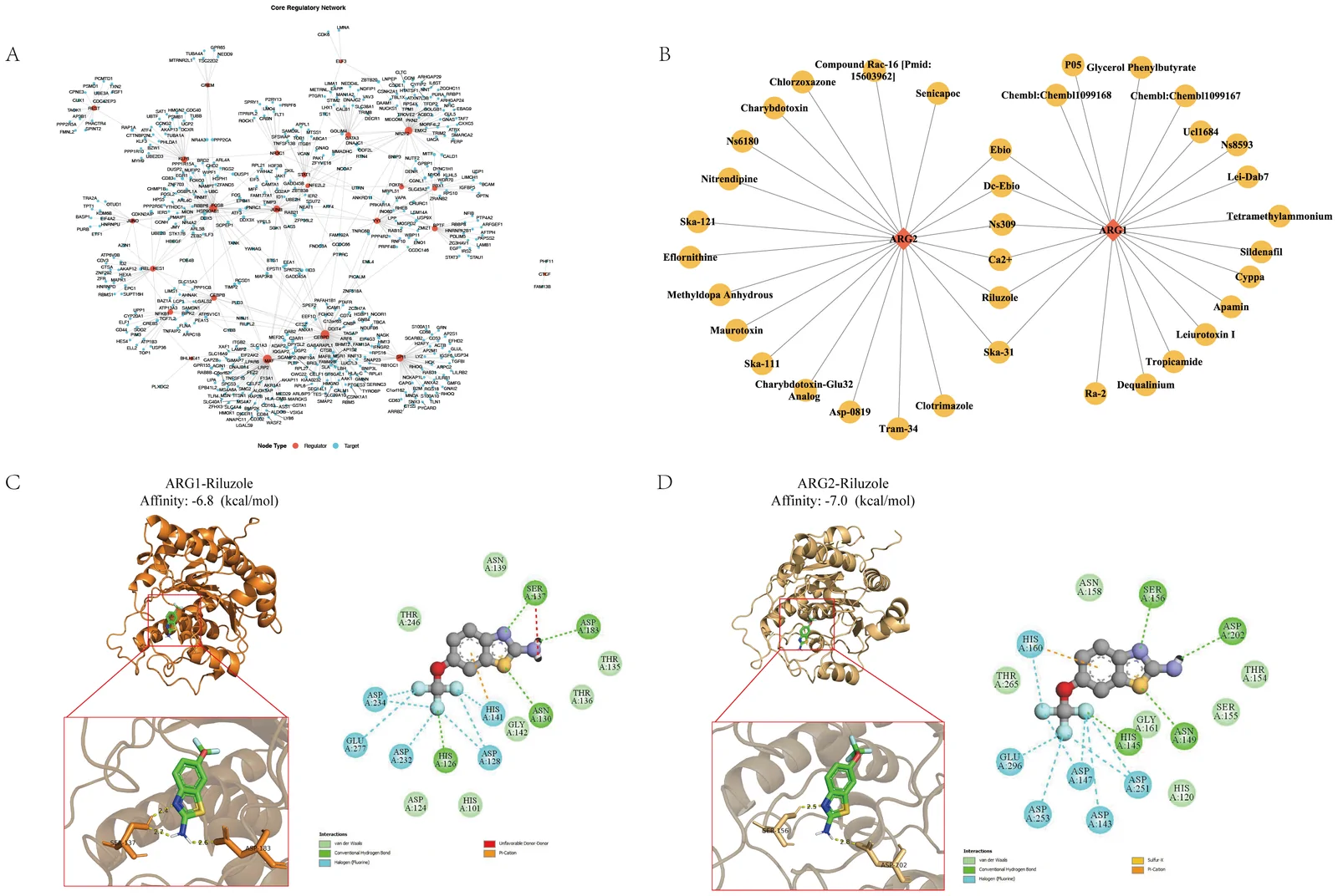
The key genes targeted by TFs and drugs. **(A)** Transcription factor (TF)-mRNA regulatory network. TFs predicted to regulate ARG1 and ARG2 (red nodes) are shown as rhombus nodes, with edges indicating regulatory relationships. **(B)** Drug-biomarker interaction network showing drugs targeting ARG1 and ARG2 identified from the DGIdb database. **(C)** Molecular docking of riluzole to ARG1. Hydrogen bonds and hydrophobic interactions are indicated. The binding energy was -6.8 kcal/mol. **(D)** Molecular docking of riluzole to ARG2. The binding energy was -7.0 kcal/mol.

In the TF-mRNA network (**Figure 6A**), ARG1 was regulated by 9 TFs, and ARG2 was regulated by 7 TFs. Among them, the regulatory factors FOXC1 and YY1 simultaneously regulated ARG1 and ARG2, and were core regulatory TFs. This co-targeting indicated that these TFs might have coordinated roles in regulating ARG1 and ARG2 expression, highlighting their potential importance in the regulatory network underlying disease pathogenesis.

Potential drugs targeting the key genes for IgAN were identified using the DGIdb database (**Figure 6B**). A total of 21 drugs targeting ARG1 and 21 drugs targeting ARG2 were found. It was noteworthy that ARG1 and ARG2 collectively predicted the drugs EBIO, SKA-31, riluzole, Ca2+, DC-EBIO, and NS309. Therefore, riluzole, an FDA-approved drug, was selected as the key drug for molecular docking analysis. According to the molecular docking results, the docking binding energies of the ARG1-riluzole and ARG2-riluzole were all less than -5 kcal/mol (**Table 1**). The specific binding situation of the ARG1-riluzole and ARG2-riluzole were shown in **Figures 6C, D**. These computational docking results suggest that riluzole may serve as a potential candidate compound for future investigation in IgAN, although further *in vitro* and *in vivo* studies are required to confirm its actual therapeutic efficacy.

**Table 1:** Molecular docking.

Pubchem_ID	MolName	Symbol	PDB_ID	Affinity(kcal/mol)
CID5070	Riluzole	ARG1	1WVA	-6.8
CID5070	Riluzole	ARG2	4HZE	-7.0

### 3.7 Monocytes identified as the key cells

**Figure 7:**
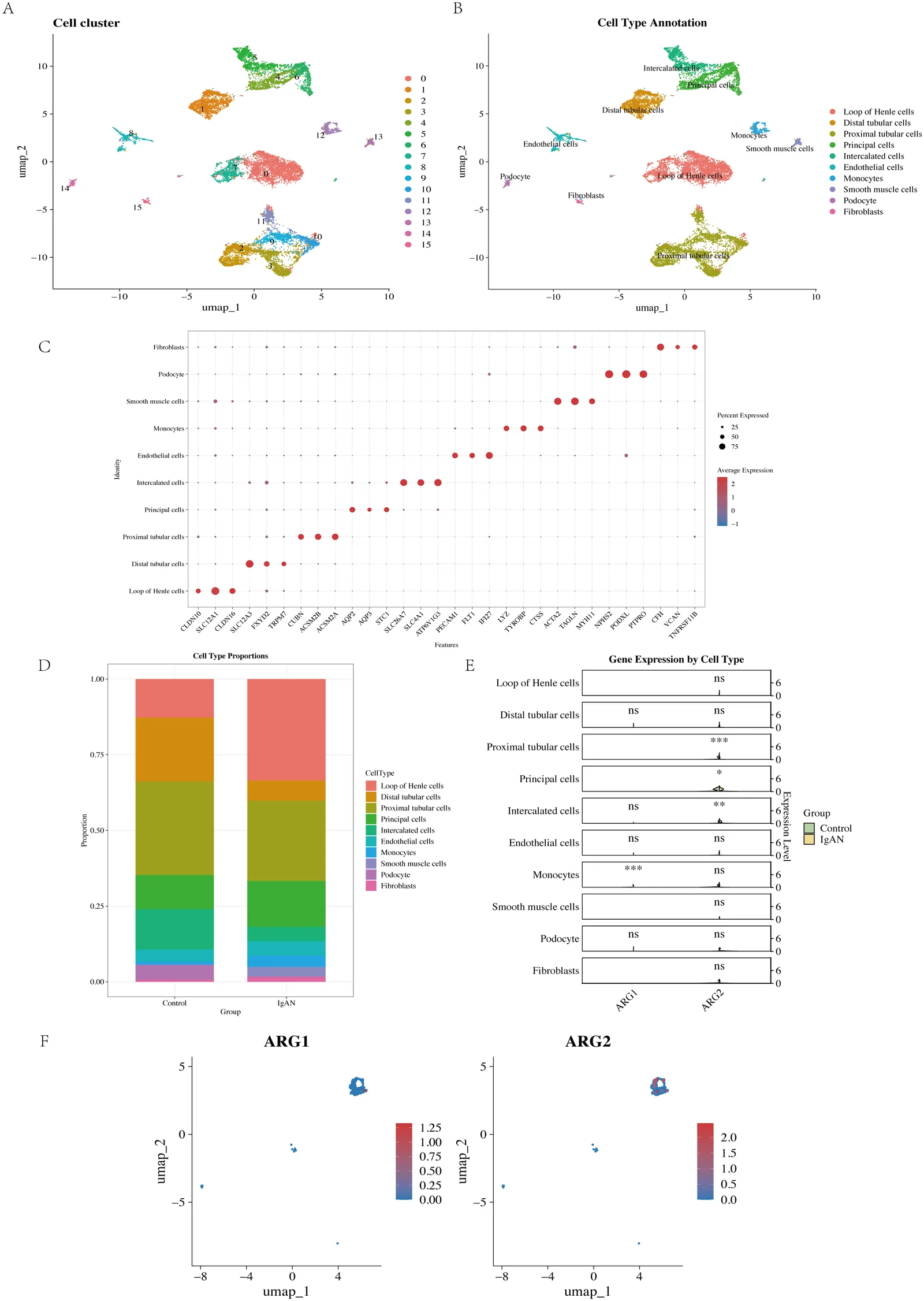
Monocytes identified as the key cells. **(A)** Uniform Manifold Approximation and Projection (UMAP) plot of 16 cell clusters identified from the GSE171314 single-cell dataset. Each dot represents an individual cell, and different colors indicate different clusters (Clusters 0–15). **(B)** UMAP plot of all single cells with cell-type annotations. Cells are colored according to cell type (10 cell types). **(C)** Scatter plot showing the expression distribution of the top marker genes used for cell type annotation across the 10 cell types. Each dot represents a cell cluster, and gene names are labeled. **(D)** Histogram showing the percentage of each cell type in IgAN and control samples. **(E)** Expression of ARG1 and ARG2 across all major cell types in IgAN and control samples. **(F)** UMAP plots showing the expression distribution of ARG1 (left) and ARG2 (right) across all single cells. Red indicates high expression, and blue indicates low expression.

First, prior to quality control of the GSE171314 dataset, the initial count comprised 19,830 cells and 26,398 genes. Subsequent to quality control, the cell count was refined to 18,733, while the gene count remained constant at 26,398 (**Supplementary Figure 1A**). Second, the top 2,000 HVGs were identified, and the top 10 genes with the greatest variability included KLK1, IL1RL1, and NPHS2 (**Supplementary Figure 1B**). After 25 PCs, the significance decreased, and the curve in the PC scree plot flattened at PC = 25. Therefore, the top 25 PCs were selected for further analysis (**Supplementary Figure 1C**). After that, cells were partitioned into 16 cell clusters using the UMAP clustering method (**Figure 7A**). Relying on the expression of marker genes (**Figure 7B**), the 16 cell clusters were annotated into 10 cell types: loop of Henle cells, distal tubular cells, proximal tubular cells, principal cells, intercalated cells, endothelial cells, monocytes, smooth muscle cells, podocytes, and fibroblasts (**Figure 7C**). As shown in the histogram, loop of Henle cells, proximal tubular cells, and principal cells were among the most abundant cell types in IgAN samples, whereas proximal tubular cells, distal tubular cells, and intercalated cells were the predominant populations in control samples (**Figure 7D**). Furthermore, in both IgAN and control samples, the ARG1 gene was significantly differentially expressed in monocytes, while the ARG2 gene was significantly differentially expressed in proximal tubular cells, principal cells, and intercalated cells (P < 0.05) (**Figure 7E**). As shown in **Figure 7F**, both ARG1 and ARG2 were predominantly expressed in monocytes, supporting their selection as the key cell type for subsequent analysis. Notably, ARG2 exhibited differential expression across multiple tubular cell subtypes, suggesting that, in addition to its immunomodulatory roles, it may also affect renal tubular epithelial cell function. This finding provided new clues for understanding the multi-cellular involvement of ARG2 in IgAN and warranted further investigation. It should be noted that the GSE171314 dataset contains only one control sample; therefore, the single-cell-based comparisons between IgAN and control samples, including cell proportion analysis, intercellular communication, and pseudotime trajectory inference, are exploratory and descriptive in nature. The findings derived from these analyses are preliminary and should be interpreted with appropriate caution. Nevertheless, they provide valuable initial insights into the cellular landscape of IgAN and generate hypotheses that warrant further validation in larger single-cell cohorts.

### 3.8 Exploring the interactions, differentiation, and upstream regulation of monocytes

**Figure 8:**
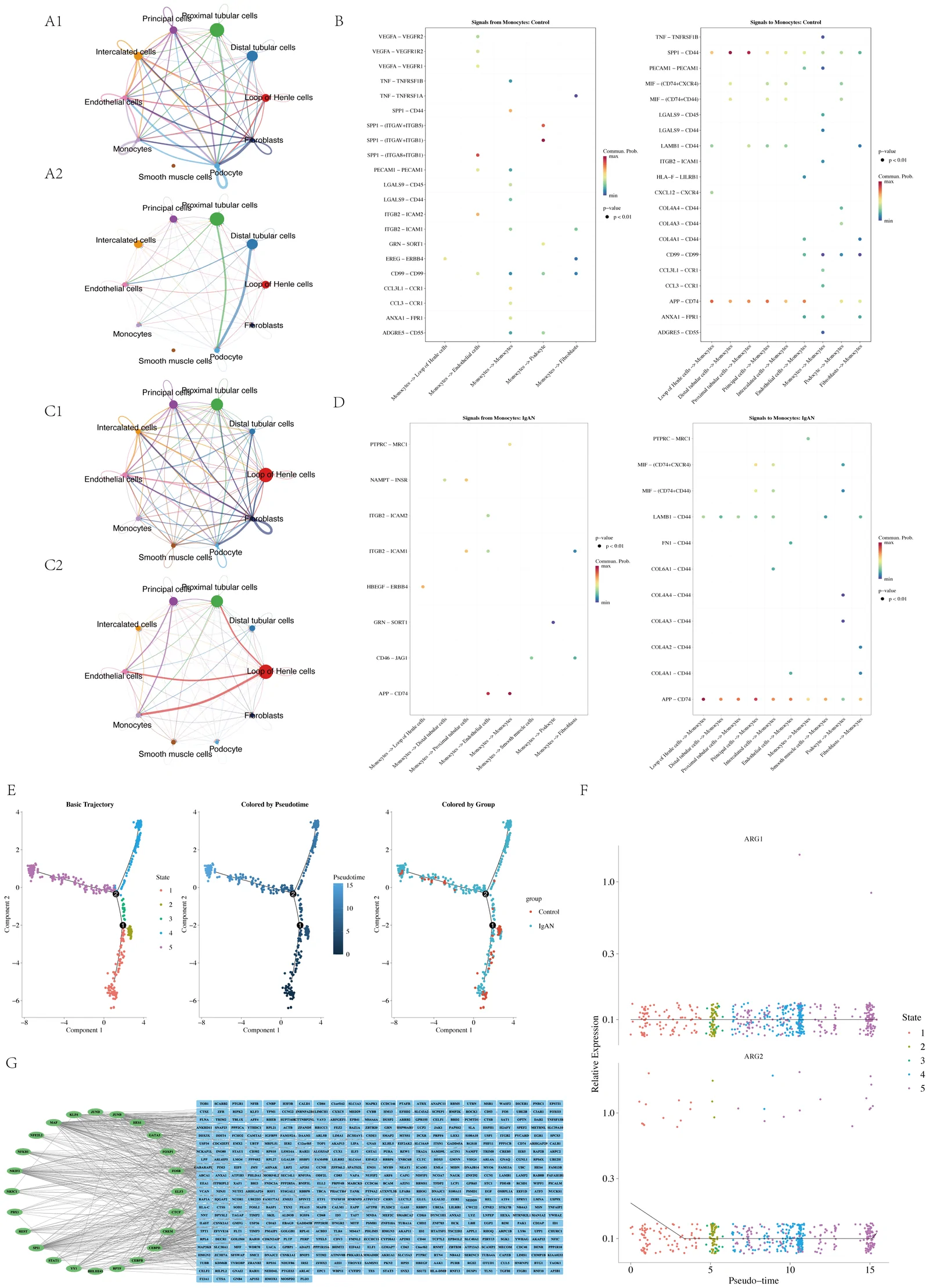
Exploring the interactions, differentiation, and upstream regulation of monocytes. **(A)** Cell-cell communication network in control samples showing the number of interactions between different cell types. Node size represents the number of outgoing/incoming interactions, and edge thickness represents the communication strength. **(B)** Bubble plot showing ligand-receptor (L-R) interactions between monocytes and other cell types in control samples. The circle size represents the contribution score of each L-R pair, and color intensity represents the communication probability. **(C)** Cell-cell communication network in IgAN samples showing the number of interactions between different cell types. **(D)** Bubble plot showing L-R interactions between monocytes and other cell types in IgAN samples. **(E)** Pseudotime analysis of monocyte differentiation. Left: Monocyte subpopulations projected onto the pseudotime trajectory, colored by 5 differentiation states (dark blue: early, light blue: mature). Right: IgAN (blue) and control (red) samples are projected onto the same pseudotime trajectory. **(F)** Expression dynamics of ARG1 and ARG2 along the monocyte pseudotime trajectory. ARG1 shows uniformly low expression across all states, while ARG2 shows low expression in most cells but high expression in a small subset. **(G)** SCENIC-derived transcription factor (TF)-target gene regulatory network in monocytes. TFs and their target genes are shown, with edge thickness representing regulatory strength. The network includes 24 TFs and 491 target genes.

The CellChat analysis suggested potential alterations in intercellular communication in IgAN samples compared with control samples, although these findings remain exploratory due to the limited sample size of the single-cell dataset. Among control samples, the number of interactions between podocytes and fibroblasts was the highest, while the interaction intensity between podocytes and distal tubular cells was the strongest. Except for smooth muscle cells, monocytes and all other cells were involved in interactions (**Figure 8A**). When monocytes acted as ligands and receptors respectively, the SPP1-(ITGAV+ITGB1) pair and SPP1-CD44 pair served as the key signaling pairs between monocytes and podocytes, and between distal tubular cells and monocytes, respectively (**Figure 8B**). For IgAN samples, the number of interactions between podocytes and fibroblasts remained the highest, while the interaction intensity between monocytes and loop of Henle cells was the strongest (**Figure 8C**). When monocytes acted as ligands, the APP-CD74 pair was the key signaling pair between monocytes and endothelial cells, while when monocytes served as receptors, the APP-CD74 pair was also the key signaling pair between loop of Henle cells and monocytes (**Figure 8D**). These findings suggested distinct signaling mechanisms underlying cell communication in different disease contexts.

Pseudotime analysis of monocytes differentiation revealed a progressive trajectory from an early (dark blue) to a more mature (light blue) state, and the subpopulations could be roughly divided into 5 differentiation states (**Figure 8E**). Notably, IgAN and control samples exhibited distinct differentiation patterns: IgAN samples displayed a continuous differentiation trajectory from early to late stages, whereas control samples were predominantly enriched in the early differentiation stage. This suggests that monocytes in IgAN undergo more extensive differentiation, potentially contributing to the pathogenesis of IgAN. Regarding the expression patterns of ARG1 and ARG2 during monocyte differentiation, we observed markedly distinct distribution characteristics between the two genes. ARG1 exhibited uniformly low expression across all monocyte subsets throughout the differentiation trajectory, suggesting that ARG1 may function as a housekeeping metabolic regulator in monocytes rather than participating in differentiation state-specific regulation. In contrast, ARG2 displayed low expression in the majority of cells but showed high expression in a small subset of cells, and this high-expression pattern was independent of the pseudotime trajectory. This expression pattern suggests that ARG2 may play a specialized role in specific monocyte functional states, possibly related to distinct monocyte subsets or activation states. Furthermore, the distinct expression patterns of ARG1 and ARG2 imply that these two arginase isoforms may be regulated by different transcriptional mechanisms in monocytes (**Figure 8F**). Finally, in the monocyte regulatory network, a total of 24 transcriptional regulatory factors (such as CEBPD, MAF, FOSB) and their corresponding 491 target genes (such as TOB1, SCARB2, CTSZ) were identified (**Figure 8G**). All the above results provided a reference for the specific mechanism of monocytes in IgAN.

### 3.9 qPCR validation of ARG1 and ARG2 expression levels in kidney tissue

**Figure 9:**
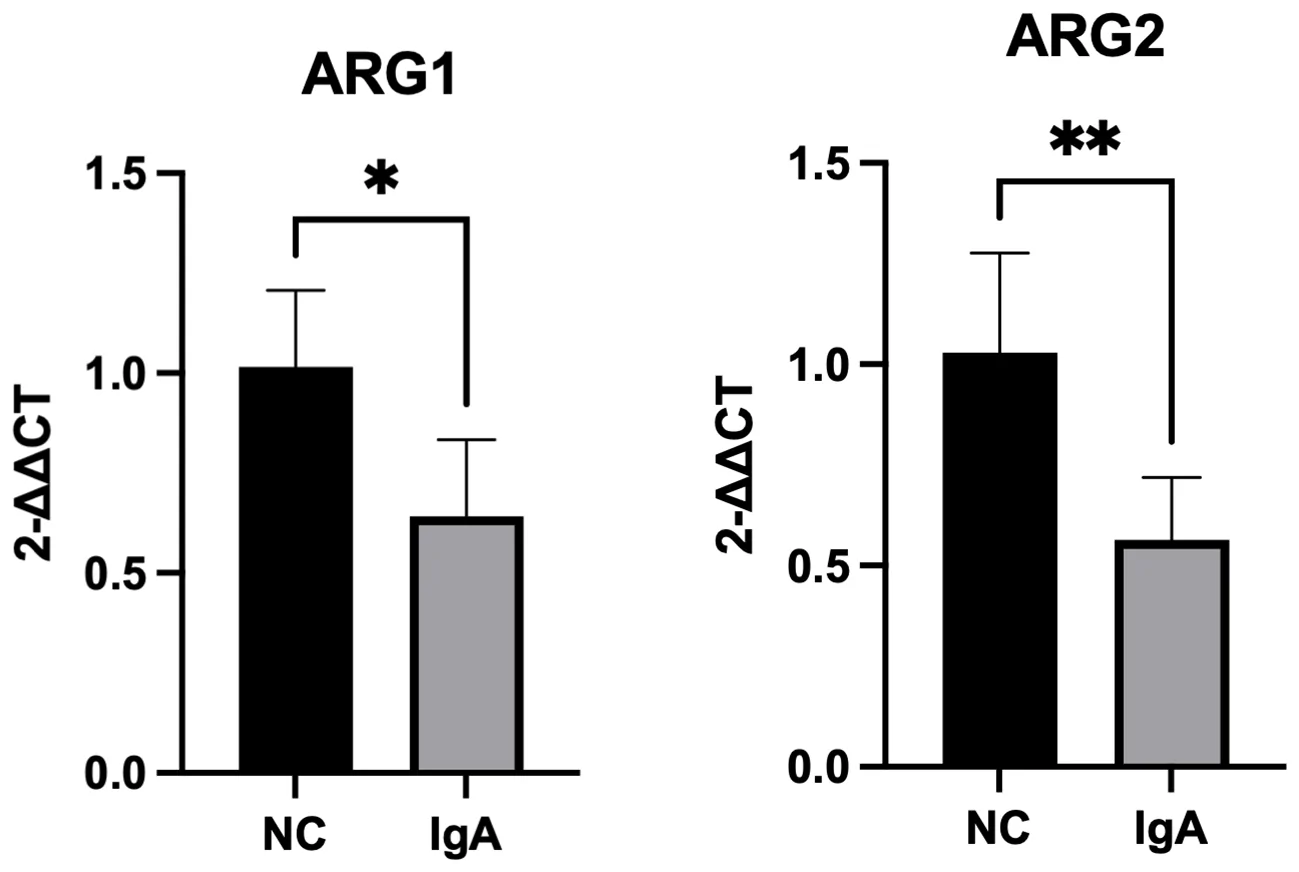
qPCR results for detecting ARG1 and ARG2 mRNA expression levels. Relative mRNA expression levels of ARG1 and ARG2 in renal tissue samples from IgAN patients (n = 5) and healthy control subjects (n = 5), normalized to GAPDH and calculated using the 2^−ΔΔCt^ method. The data are presented as mean ± standard deviation (SD) from three independent experimental replicates. IgAN patients showed significantly reduced expression of both ARG1 and ARG2 compared to the control group. *P < 0.05, **P < 0.01 (Mann–Whitney U test).

To verify the reliability of ARG1 and ARG2 as key genes, we collected renal tissue specimens from 5 patients diagnosed with IgA nephropathy via renal biopsy, and used adjacent normal renal tissue from 5 patients with renal cell carcinoma as controls. Real-time quantitative PCR was used to detect the mRNA expression levels of ARG1 and ARG2 in both groups. The results showed that, compared with the control group, the expression levels of both ARG1 and ARG2 in the renal tissue of IgA nephropathy patients were significantly lower than those in the control group (p < 0.05) (**Figure 9**).

## 4 Discussion

IgAN is the most common primary glomerulonephritis globally. Current management is mainly symptomatic and supportive. Novel targeted therapies can slow disease progression, but the relationship between recurrence, inflammation, and long-term prognosis is unclear, and the ESRD risk is high. There’s an urgent need to clarify pathogenesis and find new targets. The arginine metabolism (AM) pathway is crucial for amino acid metabolism in kidney disease. High ARG2 expression in renal tubular epithelial cells implies a key regulatory role in the kidney, yet its link with IgAN is unclear. This study analyzed scRNA-seq and bulk RNA-seq data and identified ARG1 and ARG2 as IgAN-related arginine metabolism key genes. Functional enrichment analysis showed these key genes were enriched in multiple signaling pathways like B-cell PIP3 and B-cell receptor (BCR) pathways. Most differentially expressed immune cells had a significant negative correlation with these key genes. Drug binding predictions indicated riluzole has strong binding affinity for these key genes and can form stable complexes. Also, scRNA-seq analysis showed monocytes are key cells in IgAN onset and progression.

ARG1 and ARG2 belong to the same arginase family and both catalyze arginine hydrolysis to ornithine and urea, but their functions and expression sites differ (44, 45). ARG1 is mainly in the liver, regulatory T cells, and monocytes, inhibiting immune cell over-activation by depleting local arginine (46); ARG2 is mainly in renal tubular epithelial cells and B cells, regulating intracellular arginine homeostasis and renal repair (47), and they synergistically regulate arginine metabolic balance. Arginase and iNOS use arginine as a substrate, creating metabolic competition: the arginase pathway produces ornithine for basement membrane synthesis, and the iNOS pathway produces NO for regulating inflammatory responses (7). It is conceivable that the down-regulation of ARG1 and ARG2 may disrupt the balance of arginine metabolism, potentially leading to local arginine accumulation. In B cells, accumulated arginine has been reported to activate the PIP3 signaling pathway and subsequently mTOR (48, 49). which might contribute to abnormalities in B-cell receptor signaling (50), and promote Gd-IgA1 production (51). Additionally, reduced arginase activity could compromise ornithine production, potentially impairing basement membrane integrity and filtration function. Deposited Gd-IgA1 may activate complement and recruit inflammatory cells, further degrading the basement membrane (52, 53). Collectively, these interconnected events suggest a possible mechanistic cascade linking arginase down-regulation to B-cell hyperactivity, pathogenic Gd-IgA1 production, and basement membrane injury, which may together facilitate the progression of IgAN. However, these mechanistic inferences are derived from correlative analyses and computational predictions, and therefore require direct experimental validation in future studies.

Furthermore, the biomarker-based receiver operating characteristic (ROC) curve developed in this study demonstrated excellent predictive performance for the disease, with an area under the curve (AUC) exceeding 0.99, which is higher than the AUC levels of most previous predictive models for IgA nephropathy (54, 55). This further confirms that the arginine metabolism-related receiver operating characteristic curve developed in this study demonstrates superior efficacy in the diagnosis and prediction of IgA nephropathy, and validates the reliability and clinical application potential of ARG1 and ARG2 as predictive key genes for IgA nephropathy.

To verify the reliability of the bioinformatics analysis results from public databases, this study further collected kidney tissue specimens from patients with IgA nephropathy and used qPCR to detect the mRNA expression levels of ARG1 and ARG2. The results showed that, compared with the control group, the expression of both ARG1 and ARG2 in the kidney tissue of patients with IgA nephropathy was significantly downregulated. This result is consistent with the analysis results from public databases, further enhancing the credibility of the research conclusions. ARG1 and ARG2 are key enzymes in the arginine metabolic pathway, and their abnormal expression may be involved in the pathological processes of IgA nephropathy, such as local immune inflammatory responses, metabolic imbalances, and renal interstitial fibrosis (46, 47). Therefore, ARG1 and ARG2 not only hold promise as potential molecular markers for IgA nephropathy but may also provide a theoretical basis for further elucidating the mechanisms of disease development and exploring new intervention targets. However, this study has some limitations. First, the experimental validation was performed using renal tissue samples rather than non-invasive specimens such as serum, plasma, urine, or peripheral blood cells. Therefore, the present findings cannot yet demonstrate that ARG1 or ARG2 can directly replace renal biopsy for the diagnosis of IgAN. A recent serum proteomic study reported decreased ARG1 expression in patients with progressive IgAN, suggesting that ARG1 may also have potential value as a circulating biomarker. However, because serum, plasma, urine, and peripheral blood cell samples were not available in the present cohort, we were unable to further validate the circulating levels of ARG1/ARG2 or their expression patterns in different blood cell subsets. Future studies with larger, prospectively collected cohorts are needed to determine whether ARG1 and ARG2 can serve as non-invasive key genes for IgAN diagnosis, disease activity assessment, or prognosis prediction.

The immune infiltration analysis results in this study show that there are 19 significantly different immune cells in IgAN, such as EM CD8 T cells, eosinophils, neutrophils, and monocytes. ARG1 and ARG2 have similar trends in their associations with these immune cells. They both have significant positive correlations with CD56 bright natural killer cells, eosinophils, neutrophils, and Th17 cells, and significant negative correlations with activated B cells and activated dendritic cells, especially with EM CD8 T cells. This indicates that these immune cells may participate in the progression of IgAN by regulating related gene expression. EM CD8 T cells play a key role in the disease progression as important effector cells and inflammatory amplifiers of IgAN kidney injury. They can migrate to sites of renal inflammation and differentiate into tissue-resident memory T cells (TRMs) that persistently mediate local tissue damage (56–58). These cells activate renal resident cells by secreting pro-inflammatory factors such as IFN-γ and TNF-α, triggering the release of chemokines and recruiting additional immune cells (59, 60), thereby forming an inflammatory cascade that promotes interstitial inflammation and fibrosis and exacerbates the course of IgAN (61, 62). In this study, ARG1 and ARG2 were downregulated in IgAN and showed a significant negative correlation with EM CD8 T cells. A plausible interpretation of this correlation is that reduced arginase activity may lead to arginine accumulation in the local microenvironment, potentially relieving the metabolic suppression of EM CD8 T cells. This might facilitate their activation, proliferation, and functional maintenance through mTOR pathway activation, thereby potentially enhancing the cytotoxic and pro-inflammatory activities of EM CD8 T cells (61, 63), and contributing to renal damage in IgAN. This proposed mechanism, however, is based on correlative evidence and remains to be tested through direct functional experiments, such as arginine supplementation or arginase inhibition assays in co-culture systems.

The results show that the free energies of association between riluzole and the key genes are mostly below -5 kcal/mol. Riluzole is a multi-target small-molecule drug initially developed for the treatment of neurodegenerative diseases due to its anti-glutamatergic properties. As research has progressed, its anti-inflammatory, anti-fibrotic, and immunomodulatory effects have been identified, expanding its application to chronic kidney disease, pain, and psychiatric disorders (64–67). Currently, its application is well-established only in amyotrophic lateral sclerosis (ALS), while in other fields, research is mostly in the basic experimental or early clinical trial stages. Its core mechanisms of action include counteracting glutamate-induced excitotoxicity, modulating ion channels, regulating anti-inflammatory and anti-fibrotic pathways, and immunomodulation (68). In clinical research on ALS, several Phase III trials have shown that this treatment can prolong patient survival and slow disease progression. It was approved by the FDA as a first-line treatment in 1995. Main adverse reactions are mild gastrointestinal reactions and abnormal liver function, and its safety is manageable (69, 70). In the chronic kidney disease field, riluzole had anti-ischemic effects in rat renal IRI models, suggesting potential therapeutic value (66), but optimal dosage and safety are unclear due to lack of large-scale clinical controlled trials. However, it should be emphasized that these molecular docking results are computational predictions and cannot establish therapeutic efficacy *in vivo*. The predicted binding affinity between riluzole and ARG1/ARG2 provides a preliminary computational basis, but rigorous *in vitro* enzyme activity assays, cellular functional studies, and *in vivo* animal models are essential before any clinical implication can be drawn. Future work should focus on validating the functional consequences of riluzole on arginase activity and its potential effects in IgAN models.

According to biomarker expression profiling analysis, monocytes are important in the pathogenesis of IgAN. As core innate immunity cells, monocytes maintain immune homeostasis by phagocytosing and clearing pathogens and debris, secreting cytokines, presenting antigens, and regulating immune responses. They can also migrate to injured tissues, differentiate into macrophages, and participate in tissue repair and remodeling. In IgAN, deposited Gd-IgA1 immune complexes activate glomerular mesangial and tubular epithelial cells, which release chemokines to recruit monocytes to the kidney. After entering renal tissue, monocytes differentiate into pro-inflammatory M1 and pro-fibrotic M2 macrophages under local microenvironment induction. Moreover, the differentiated macrophages act on CD4^+^T cells, promoting Th17 differentiation and stimulating B cells to produce more Gd-IgA1, forming a pathogenic vicious cycle that exacerbates IgAN renal inflammation and chronic injury. The cell-cell communication analysis based on the single-cell dataset revealed potentially altered signaling interactions between monocytes and other renal cell types in IgAN. In this study, monocyte infiltration was found to be increased in IgAN, and ARG1 expression was negatively correlated with monocyte infiltration. ARG1 also exhibited significant expression variations among monocytes. As a key enzyme for arginine degradation, down-regulation of ARG1 might result in local and peripheral arginine accumulation in the kidney, potentially activating the mTOR signaling pathway in monocytes. This could, in theory, promote monocyte mobilization from the bone marrow to the peripheral blood and enhance their sensitivity to chemokines at renal injury sites, thereby increasing monocyte infiltration into the kidney (71). Additionally, reduced ARG1 expression may alter the functional composition of monocyte subsets, potentially redirecting arginine metabolism toward the iNOS-NO pathway, activating NF-κB signaling, and favoring monocyte polarization toward a pro-inflammatory phenotype (72, 73). Such changes might contribute to renal inflammation and fibrosis in IgAN. Nevertheless, these mechanistic hypotheses are primarily derived from correlations observed in transcriptomic data and immune infiltration estimations, and warrant direct experimental verification using *in vitro* functional assays and *in vivo* models.

This study identified ARG1 and ARG2 as AM-associated key genes in IgAN. These genes may influence the infiltration of immune cells such as EM CD8 T cells and monocytes by participating in B-cell signaling pathways and the basement membrane, thereby affecting the onset and progression of IgAN. This offers a new theoretical basis and direction for exploring the specific mechanisms of IgAN progression related to these genes. However, the study has limitations. First, it used bioinformatics analysis to propose a theoretical diagnostic model. The preliminary investigation of biomarker expression levels needs further validation with a large number of samples to enhance the clinical translational value. Additionally, the use of datasets derived from different platforms may introduce potential batch effects. We also acknowledge that the datasets used overlap with previous bioinformatics studies on IgAN; we did not include additional datasets because publicly available kidney tissue transcriptomic cohorts with complete metadata remain limited, and adding datasets from other tissue sources would introduce heterogeneity that could confound our findings. Second, immunophenotyping and cellular infiltration analyses were based on limited genetic data, and interactions among atypical cells may introduce bias into immunological analyses. Third, regarding the single-cell dataset GSE171314, we acknowledge that: (a) it is derived from kidney tissue and predominantly contains parenchymal cell types, with monocytes representing the only clearly identifiable immune cell population, which precludes a comprehensive evaluation of ARG1 and ARG2 expression in other immune cell types such as T cells, macrophages, and dendritic cells; and (b) it includes only 4 IgAN samples and 1 control sample, limiting the statistical power for group-wise comparisons and rendering the CellChat-based cell-cell communication findings exploratory, which should be interpreted with caution and require further validation. Fourth, the diagnostic nomogram developed in this study achieved an AUC of 0.991 in the training set, which may reflect overfitting due to the limited sample size of GSE93798 (20 IgAN vs 22 controls). This high performance requires further validation in larger independent cohorts. Fifth, the qPCR validation used adjacent normal tissues from renal cell carcinoma patients as controls. It should be acknowledged that peritumoral tissues are subject to field effects, which may cause molecular alterations even when the tissue appears histologically normal, potentially affecting gene expression baselines and rendering them less than ideal healthy references. Therefore, the observed downregulation of ARG1 and ARG2 in IgAN tissues should be interpreted as a supporting and preliminary observation rather than definitive validation. The modest sample size (n = 5 per group) further limits the statistical power of this experimental confirmation. Future studies utilizing kidney tissues from truly healthy donors or larger, well-characterized independent cohorts are warranted to corroborate these findings. Sixth, the current validation is limited to the mRNA level, and protein-level evidence such as immunohistochemistry, Western blotting, or enzyme activity assays is lacking to fully confirm the biological relevance of ARG1 and ARG2 in IgAN. Finally, Western blotting and immunohistochemistry experiments are needed to fully understand the roles of these key genes and their regulatory mechanisms in IgAN.

## Data Availability

The original contributions presented in the study are included in the article/**Supplementary Material**. Further inquiries can be directed to the corresponding author.

## Supplementary material

Supplementary Figure 1 Single cell data quality control.

Supplementary Figure 2 Construction and evaluation of the validation set column chart.

Supplementary Table 1 Arginine metabolism-related genes.

Supplementary Table 2 GSVA enrichment pathways.

Supplementary Table 3 The GO terms for the candidate genes.

Supplementary Table 4 The KEGG pathways for the candidate genes.

Supplementary Table 5 Details of the bar chart for the training set and validation set.

Supplementary Table 6 GSEA enrichment pathways.

Supplementary Table 7 Raw rt-qpcr data and primer sequences.

## References

[1] Lee M Suzuki H Nihei Y Matsuzaki K Suzuki Y . Ethnicity and IgA nephropathy: worldwide differences in epidemiology, timing of diagnosis, clinical manifestations, management and prognosis. Clin Kidney J. (2023) 16:ii1–8. doi: 10.1093/ckj/sfad199 38053973 PMC10695519

[2] Stamellou E Seikrit C Tang SCW Boor P Tesař V Floege J . IgA nephropathy. Nat Rev Dis Primers. (2023) 9:67. doi: 10.1038/s41572-023-00476-9 38036542

[3] Cheung CK Barratt J Liew A Zhang H Tesar V Lafayette R . The role of BAFF and APRIL in IgA nephropathy: pathogenic mechanisms and targeted therapies. Front Nephrol. (2023) 3:1346769. doi: 10.3389/fneph.2023.1346769 38362118 PMC10867227

[4] El Labban M Surani S . Immunoglobulin a glomerulonephropathy: a review. World J Clin cases. (2024) 12:1388–94. doi: 10.12998/wjcc.v12.i8.1388 38576821 PMC10989439

[5] Zhang Z Zhang Y Zhang H . IgA nephropathy: a Chinese perspective. Glomerular Dis. (2022) 2:30–41. doi: 10.1159/000520039 36751266 PMC9677733

[6] Kamal F Kim J Lafayette R . Current biomarkers of IgA nephropathy. Semin Nephrol. (2024) 44:151572. doi: 10.1016/j.semnephrol.2025.151572 40087126

[7] Correale J . Immunosuppressive amino-acid catabolizing enzymes in multiple sclerosis. Front Immunol. (2020) 11:600428. doi: 10.3389/fimmu.2020.600428 33552055 PMC7855700

[8] Fung TS Ryu KW Thompson CB . Arginine: at the crossroads of nitrogen metabolism. EMBO J. (2025) 44:1275–93. doi: 10.1038/s44318-025-00379-3 39920310 PMC11876448

[9] Li R Li Y Jiang K Zhang L Li T Zhao A . Lighting up arginine metabolism reveals its functional diversity in physiology and pathology. Cell Metab. (2025) 37:291–304.e9. doi: 10.1016/j.cmet.2024.09.011 39413790

[10] Guan Y Yin X Wang L Diao Z Huang H Wang X . Biomarkers of arginine methylation in diabetic nephropathy: novel insights from bioinformatics analysis. Diabetes Metab Syndr Obes. (2024) 17:3399–418. doi: 10.2147/DMSO.S472412 39290792 PMC11407315

[11] Aihara S Torisu K Uchida Y Imazu N Nakano T Kitazono T . Spermidine from arginine metabolism activates Nrf2 and inhibits kidney fibrosis. Commun Biol. (2023) 6:676. doi: 10.1038/s42003-023-05057-w 37380734 PMC10307812

[12] Li M Wang L Shi DC Foo JN Zhong Z Khor CC . Genome-wide meta-analysis identifies three novel susceptibility loci and reveals ethnic heterogeneity of genetic susceptibility for IgA nephropathy. J Am Soc Nephrol. (2020) 31:2949–63. doi: 10.1681/ASN.2019080799 32912934 PMC7790208

[13] Jovic D Liang X Zeng H Lin L Xu F Luo Y . Single-cell RNA sequencing technologies and applications: a brief overview. Clin Transl Med. (2022) 12:e694. doi: 10.1002/ctm2.694 35352511 PMC8964935

[14] Shen X Mo S Zeng X Wang Y Lin L Weng M . Identification of antigen-presentation related B cells as a key player in crohn’s disease using single-cell dissecting, hdWGCNA, and deep learning. Clin Exp Med. (2023) 23:5255–67. doi: 10.1007/s10238-023-01145-7 37550553

[15] Lin Y Jing X Chen Z Pan X Xu D Yu X . Histone deacetylase-mediated tumor microenvironment characteristics and synergistic immunotherapy in gastric cancer. Theranostics. (2023) 13:4574–600. doi: 10.7150/thno.86928 37649598 PMC10465215

[16] Guan J Xu X Qiu G He C Lu X Wang K . Cellular hierarchy framework based on single-cell/multi-patient sample sequencing reveals metabolic biomarker PYGL as a therapeutic target for HNSCC. J Exp Clin Cancer Res. (2023) 42:162. doi: 10.1186/s13046-023-02734-w 37420300 PMC10329320

[17] Ritchie ME Phipson B Wu D Hu Y Law CW Shi W . limma powers differential expression analyses for RNA-sequencing and microarray studies. Nucleic Acids Res. (2015) 43:e47. doi: 10.1093/nar/gkv007 25605792 PMC4402510

[18] Gustavsson EK Zhang D Reynolds RH Garcia-Ruiz S Ryten M . ggtranscript: an R package for the visualization and interpretation of transcript isoforms using ggplot2. Bioinformatics. (2022) 38:3844–6. doi: 10.1093/bioinformatics/btac409 35751589 PMC9344834

[19] Zhang X Chao P Zhang L Xu L Cui X Wang S . Single-cell RNA and transcriptome sequencing profiles identify immune-associated key genes in the development of diabetic kidney disease. Front Immunol. (2023) 14:1030198. doi: 10.3389/fimmu.2023.1030198 37063851 PMC10091903

[20] Chen H Boutros PC . VennDiagram: a package for the generation of highly-customizable venn and euler diagrams in R. BMC Bioinf. (2011) 12:35. doi: 10.1186/1471-2105-12-35 21269502 PMC3041657

[21] Wu T Hu E Xu S Chen M Guo P Dai Z . clusterProfiler 4.0: a universal enrichment tool for interpreting omics data. Innovation (Camb). (2021) 2:100141. doi: 10.1016/j.xinn.2021.100141 34557778 PMC8454663

[22] Shannon P Markiel A Ozier O Baliga NS Wang JT Ramage D . Cytoscape: a software environment for integrated models of biomolecular interaction networks. Genome Res. (2003) 13:2498–504. doi: 10.1101/gr.1239303 14597658 PMC403769

[23] Friedman J Hastie T Tibshirani R . Regularization paths for generalized linear models via coordinate descent. J Stat Softw. (2010) 33:1–22. doi: 10.18637/jss.v033.i01 20808728 PMC2929880

[24] Cinelli M Sun Y Best K Heather JM Reich-Zeliger S Shifrut E . Feature selection using a one dimensional naïve bayes’ classifier increases the accuracy of support vector machine classification of CDR3 repertoires. Bioinformatics. (2017) 33:951–5. doi: 10.1093/bioinformatics/btw771 28073756 PMC5860388

[25] Zibibula Y Tayier G Maimaiti A Liu T Lu J . Machine learning approaches to identify the link between heavy metal exposure and ischemic stroke using the US NHANES data from 2003 to 2018. Front Public Health. (2024) 12:1388257. doi: 10.3389/fpubh.2024.1388257 39351032 PMC11439780

[26] Svetnik V Liaw A Tong C Culberson JC Sheridan RP Feuston BP . Random forest: a classification and regression tool for compound classification and QSAR modeling. J Chem Inf Comput Sci. (2003) 43:1947–58. doi: 10.1021/ci034160g 14632445

[27] Welham Z Déjean S Lê Cao KA . Multivariate analysis with the R package mixOmics. Methods Mol Biol. (2023) 2426:333–59. doi: 10.1007/978-1-0716-1967-4_15 36308696

[28] Robles-Jimenez LE Aranda-Aguirre E Castelan-Ortega OA Shettino-Bermudez BS Ortiz-Salinas R Miranda M . Worldwide traceability of antibiotic residues from livestock in wastewater and soil: a systematic review. Anim (Basel). (2021) 12:60. doi: 10.3390/ani12010060 35011166 PMC8749557

[29] Tang Q Zhou X Zhang B Ma C Zou Y Yuan Z . Integrative multi-omics and machine learning identify CALR as a diagnostic and therapeutic target in aneurysmal subarachnoid hemorrhage. Exp Neurol. (2025) 393:115396. doi: 10.1016/j.expneurol.2025.115396 40714016

[30] S L H Y Y H G X B L J L . Evaluation of the prognostic value of centrosome-related genes and assessment of the immune microenvironment in osteosarcoma. Biochem Biophys Res Commun. (2025) 786. doi: 10.1016/j.bbrc.2025.152739 41046658

[31] Robin X Turck N Hainard A Tiberti N Lisacek F Sanchez JC . pROC: an open-source package for R and S+ to analyze and compare ROC curves. BMC Bioinf. (2011) 12:77. doi: 10.1186/1471-2105-12-77 21414208 PMC3068975

[32] Goncharova IA Nazarenko MS Babushkina NP Markov AV Pecherina TB Kashtalap VV . genetic predisposition to early myocardial infarction. Mol Biol (Mosk). (2020) 54:224–32. doi: 10.31857/S0026898420020044 32392191

[33] Hänzelmann S Castelo R Guinney J . GSVA: gene set variation analysis for microarray and RNA-seq data. BMC Bioinf. (2013) 14:7. doi: 10.1186/1471-2105-14-7 23323831 PMC3618321

[34] Liu ZY Huang RH . Integrating single-cell RNA-sequencing and bulk RNA-sequencing data to explore the role of mitophagy-related genes in prostate cancer. Heliyon. (2024) 10:e30766. doi: 10.1016/j.heliyon.2024.e30766 38774081 PMC11107114

[35] Rosignoli S Paiardini A . Boosting the full potential of PyMOL with structural biology plugins. Biomolecules. (2022) 12:1764. doi: 10.3390/biom12121764 36551192 PMC9775141

[36] Seeliger D de Groot BL . Ligand docking and binding site analysis with PyMOL and autodock/vina. J Comput-Aided Mol Des. (2010) 24:417–22. doi: 10.1007/s10822-010-9352-6 20401516 PMC2881210

[37] Trott O Olson AJ . AutoDock vina: improving the speed and accuracy of docking with a new scoring function, efficient optimization, and multithreading. J Comput Chem. (2010) 31:455–61. doi: 10.1002/jcc.21334 19499576 PMC3041641

[38] Satija R Farrell JA Gennert D Schier AF Regev A . Spatial reconstruction of single-cell gene expression data. Nat Biotechnol. (2015) 33:495–502. doi: 10.1038/nbt.3192 25867923 PMC4430369

[39] Tang R Meng T Lin W Shen C Ooi JD Eggenhuizen PJ . A partial picture of the single-cell transcriptomics of human IgA nephropathy. Front Immunol. (2021) 12:645988. doi: 10.3389/fimmu.2021.645988 33936064 PMC8085501

[40] Jin S Guerrero-Juarez CF Zhang L Chang I Ramos R Kuan CH . Inference and analysis of cell-cell communication using CellChat. Nat Commun. (2021) 12:1088. doi: 10.1038/s41467-021-21246-9 33597522 PMC7889871

[41] Hong B Li Y Yang R Dai S Zhan Y Zhang WB . Single-cell transcriptional profiling reveals heterogeneity and developmental trajectories of ewing sarcoma. J Cancer Res Clin Oncol. (2022) 148:3267–80. doi: 10.1007/s00432-022-04073-3 35713707 PMC11800893

[42] Van de Sande B Flerin C Davie K De Waegeneer M Hulselmans G Aibar S . A scalable SCENIC workflow for single-cell gene regulatory network analysis. Nat Protoc. (2020) 15:2247–76. doi: 10.1038/s41596-020-0336-2 32561888

[43] Deng Y Zheng Y Li D Hong Q Zhang M Li Q . Expression characteristics of interferon-stimulated genes and possible regulatory mechanisms in lupus patients using transcriptomics analyses. EBioMedicine. (2021) 70:103477. doi: 10.1016/j.ebiom.2021.103477 34284174 PMC8318865

[44] Niu F Yu Y Li Z Ren Y Li Z Ye Q . Arginase: an emerging and promising therapeutic target for cancer treatment. BioMed Pharmacother. (2022) 149:112840. doi: 10.1016/j.biopha.2022.112840 35316752

[45] Heuser SK Li J Pudewell S LoBue A Li Z Cortese-Krott MM . Biochemistry, pharmacology, and *in vivo* function of arginases. Pharmacol Rev. (2025) 77:100015. doi: 10.1124/pharmrev.124.001271 39952693 PMC12105760

[46] Detroja TS Samson AO . Virtual screening for FDA-approved drugs that selectively inhibit arginase type 1 and 2. Molecules. (2022) 27:5134. doi: 10.3390/molecules27165134 36014374 PMC9416497

[47] Ren Y Li Z Li W Fan X Han F Huang Y . Arginase: biological and therapeutic implications in diabetes mellitus and its complications. Oxid Med Cell Longev. (2022) 2022:2419412. doi: 10.1155/2022/2419412 36338341 PMC9629921

[48] Ge Y Li F He Y Cao Y Guo W Hu G . L-arginine stimulates the proliferation of mouse mammary epithelial cells and the development of mammary gland in pubertal mice by activating the GPRC6A/PI3K/AKT/mTOR signalling pathway. J Anim Physiol Anim Nutr (Berl). (2022) 106:1383–95. doi: 10.1111/jpn.13730 35616019

[49] Gao Z Xue M Wang Z . LC-MS/MS assay to confirm that the endogenous metabolite L-arginine promotes trophoblast invasion in the placenta accreta spectrum through upregulation of the GPRC6A/PI3K/AKT pathway. BMC Pregnancy Childbirth. (2025) 25:402. doi: 10.1186/s12884-025-07475-6 40197285 PMC11977930

[50] Lu W Skrzypczynska KM Weiss A . Acute csk inhibition hinders B cell activation by constraining the PI3 kinase pathway. Proc Natl Acad Sci USA. (2021) 118:e2108957118. doi: 10.1073/pnas.2108957118 34675079 PMC8639343

[51] Gan Y Li J Li W Han Q Zhang R Yu H . Dynamics of chromatin accessibility governing gd-IgA1 synthesis in B cells associated with IgA nephropathy. Exp Mol Med. (2025) 57:1593–606. doi: 10.1038/s12276-025-01505-1 40702134 PMC12322228

[52] Zan J Liu L Li G Zheng H Chen N Wang C . Effect of telitacicept on circulating gd-IgA1 and IgA-containing immune complexes in IgA nephropathy. Kidney Int Rep. (2024) 9:1067–71. doi: 10.1016/j.ekir.2024.01.003 38765591 PMC11101733

[53] Juan YT Chiang WC Lin WC Yang CW Chou SF Hung RW . Associations between biomarkers of complement activation, galactose-deficient IgA1 antibody and the updated oxford pathology classification of IgA nephropathy. J Clin Med. (2022) 11:4231. doi: 10.3390/jcm11144231 35887995 PMC9323307

[54] Wang Y Sun N He R Wang Z Jin J Gao T . Molecular characterization of m6A RNA methylation regulators with features of immune dysregulation in IgA nephropathy. Clin Exp Med. (2024) 24:92. doi: 10.1007/s10238-024-01346-8 38693353 PMC11062981

[55] Zhang Y Wang Z Tang W Yuan X Xie X . Development and internal and external validation of a nomogram model for predicting the risk of chronic kidney disease progression in IgA nephropathy patients. PeerJ. (2024) 12:e18416. doi: 10.7717/peerj.18416 39494280 PMC11531260

[56] Li L Tang W Zhang Y Jia M Wang L Li Q . Targeting tissue-resident memory CD8+ T cells in the kidney is a potential therapeutic strategy to ameliorate podocyte injury and glomerulosclerosis. Mol Ther. (2022) 30:2746–59. doi: 10.1016/j.ymthe.2022.04.024 35514086 PMC9372318

[57] Lai Y Zhuang L Zhu J Wang S Guo C Chen B . Novel approach to alleviate lupus nephritis: Targeting the NLRP3 inflammasome in CD8+CD69+CD103+ TRM cells. J Transl Med. (2024) 22:1139. doi: 10.1186/s12967-024-05951-9 39716284 PMC11668076

[58] Doan Ngoc TM Tilly G Danger R Bonizec O Masset C Guérif P . Effector memory-expressing CD45RA (TEMRA) CD8+ T cells from kidney transplant recipients exhibit enhanced purinergic P2X4 receptor-dependent proinflammatory and migratory responses. J Am Soc Nephrol. (2022) 33:2211–31. doi: 10.1681/ASN.2022030286 36280286 PMC9731633

[59] Franken A Van Mol P Vanmassenhove S Donders E Schepers R Van Brussel T . Single-cell transcriptomics identifies pathogenic T-helper 17.1 cells and pro-inflammatory monocytes in immune checkpoint inhibitor-related pneumonitis. J Immunother Cancer. (2022) 10:e005323. doi: 10.1136/jitc-2022-005323 36171010 PMC9528720

[60] Liu D Badell IR Ford ML . Selective CD28 blockade attenuates CTLA-4-dependent CD8+ memory T cell effector function and prolongs graft survival. JCI Insight. (2018) 3:e96378, 96378. doi: 10.1172/jci.insight.96378 29321374 PMC5821191

[61] Banu N Mozes MM Kopp JB Ziyadeh FN Meyers CM . Regulation of inducible class II MHC, costimulatory molecules, and cytokine expression in TGF-beta1 knockout renal epithelial cells: Effect of exogenous TGF-beta1. Exp Nephrol. (2002) 10:320–31. doi: 10.1159/000065295 12381916

[62] Lind HT Hall SC Strait AA Goon JB Aleman JD Chen SMY . MHC class I upregulation contributes to the therapeutic response to radiotherapy in combination with anti-PD-L1/anti-TGF-β in squamous cell carcinomas with enhanced CD8 T cell memory-driven response. Cancer Lett. (2025) 608:217347. doi: 10.1016/j.canlet.2024.217347 39580046 PMC11875078

[63] Glöckner HJ Martinenaite E Landkildehus Lisle T Grauslund J Ahmad S Met Ø . Arginase-1 specific CD8+ T cells react toward Malignant and regulatory myeloid cells. Oncoimmunology. (2024) 13:2318053. doi: 10.1080/2162402X.2024.2318053 38404966 PMC10885169

[64] Al-Horani RA . Riluzole and its prodrugs for the treatment of alzheimer’s disease. Pharm Pat Anal. (2023) 12:79–85. doi: 10.4155/ppa-2023-0001 37140357 PMC10318568

[65] Fehlings MG Pedro KM Alvi MA Badhiwala JH Ahn H Farhadi HF . Riluzole for degenerative cervical myelopathy: A secondary analysis of the CSM-PROTECT trial. JAMA Netw Open. (2024) 7:e2415643. doi: 10.1001/jamanetworkopen.2024.15643 38904964 PMC11193126

[66] Deng Y Li RW Yang YL Weiss S Smith PN . Pharmacological prevention of renal ischemia-reperfusion injury in a rat model. Anz J Surg. (2022) 92:518–25. doi: 10.1111/ans.17381 34820987

[67] Kawashima Y Yamada M Furuie H Kuniishi H Akagi K Kawashima T . Effects of riluzole on psychiatric disorders with anxiety or fear as primary symptoms: A systematic review. Neuropsychopharmacol Rep. (2023) 43:320–7. doi: 10.1002/npr2.12364 37463744 PMC10496048

[68] Blyufer A Lhamo S Tam C Tariq I Thavornwatanayong T Mahajan SS . Riluzole: A neuroprotective drug with potential as a novel anti- cancer agent (review). Int J Oncol. (2021) 59:95. doi: 10.3892/ijo.2021.5275 34713302 PMC8562386

[69] Tzeplaeff L Wilfling S Requardt MV Herdick M . Current state and future directions in the therapy of ALS. Cells. (2023) 12:1523. doi: 10.3390/cells12111523 37296644 PMC10252394

[70] Rokade AV Yelne P Giri A . Riluzole and edavarone: The hope against amyotrophic lateral sclerosis. Cureus. (2022) 14:e30035. doi: 10.7759/cureus.30035 36381733 PMC9637445

[71] Vegting Y Jongejan A Neele AE Claessen N Sela G Prange KHM . Infiltrative classical monocyte-derived and SPP1 lipid-associated macrophages mediate inflammation and fibrosis in ANCA-associated glomerulonephritis. Nephrol Dial Transplant. (2025) 40:1416–27. doi: 10.1093/ndt/gfae292 39694817

[72] Li X Wang Z Chen Y Yang Y Shao H Feng X . Regulation of monocyte polarization through nuclear factor kappa B /inhibitor of kappa B alpha pathway by cuscuta chinensis lam. In postmenopausal osteoporosis. J Ethnopharmacol. (2025) 346:119710. doi: 10.1016/j.jep.2025.119710 40154899

[73] Wang ZQ Chen MT Zhang R Zhang Y Li W Li YG . Docosahexaenoic acid attenuates doxorubicin-induced cytotoxicity and inflammation by suppressing NF-κB/iNOS/NO signaling pathway activation in H9C2 cardiac cells. J Cardiovasc Pharmacol. (2016) 67:283–9. doi: 10.1097/FJC.0000000000000350 26657886

